# Iron Sulfide Microspheres Supported on Cellulose-Carbon Nanotube Conductive Flexible Film as an Electrode Material for Aqueous-Based Symmetric Supercapacitors with High Voltage

**DOI:** 10.1021/acsomega.4c03232

**Published:** 2024-06-06

**Authors:** Jincy Parayangattil Jyothibasu, You-Ching Tien, Zi-Ting Chen, Hongta Yang, Tzu Hsuan Chiang, Ahmed F. M. EL-Mahdy, Rong-Ho Lee

**Affiliations:** † Department of Chemical Engineering, National Chung Hsing University, Taichung 402, Taiwan; ‡ Department of Energy Engineering, National United University, Miaoli 360302, Taiwan; § Department of Materials and Optoelectronic Science, National Sun Yat-Sen University, Kaohsiung 80424, Taiwan; ∥ Department of Chemical Engineering and Materials Science, Yuan Ze University, Taoyuan 320, Taiwan

## Abstract

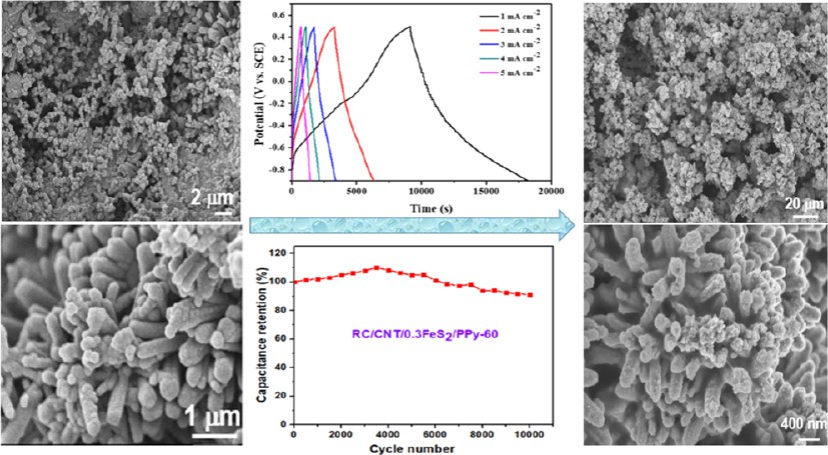

Nanostructured iron disulfide (FeS_2_) was uniformly deposited on regenerated cellulose (RC) and oxidized carbon nanotube (CNT)-based composite films using a simple chemical bath deposition method to form RC/CNT/FeS_2_ composite films. The RC/CNT composite film served as an ideal substrate for the homogeneous deposition of FeS_2_ microspheres due to its unique porous architecture, large specific surface area, and high conductivity. Polypyrrole (PPy), a conductive polymer, was coated on the RC/CNT/FeS_2_ composite to improve its conductivity and cycling stability. Due to the synergistic effect of FeS_2_ with high redox activity and PPy with high stability and conductivity, the RC/CNT/FeS_2_/PPy composite electrode exhibited excellent electrochemical performance. The RC/CNT/0.3FeS_2_/PPy-60 composite electrode tested with Na_2_SO_4_ aqueous electrolyte could achieve an excellent areal capacitance of 6543.8 mF cm^–2^ at a current density of 1 mA cm^–2^. The electrode retained 91.1% of its original capacitance after 10,000 charge/discharge cycles. Scanning electron microscopy (SEM) images showed that the ion transfer channels with a pore diameter of 5–30 μm were formed in the RC/CNT/0.3FeS_2_/PPy-60 film after a 10,000 cycle test. A symmetrical supercapacitor device composed of two identical pieces of RC/CNT/0.3FeS_2_/PPy-60 composite electrodes provided a high areal capacitance of 1280 mF cm^–2^, a maximum energy density of 329 μWh cm^–2^, a maximum power density of 24.9 mW cm^–2^, and 86.2% of capacitance retention after 10,000 cycles at 40 mA cm^–2^ when tested at a wide voltage window of 1.4 V. These results demonstrate the greatest potential of RC/CNT/FeS_2_/PPy composite electrodes for the fabrication of high-performance symmetric supercapacitors with high operating voltages.

## 1 Introduction

Modern society is highly dependent on energy, which is also essential for the development of human civilization. Due to the rapid depletion of fossil fuels, environmental pollution, and global warming, it is critical to explore renewable and clean energy sources and develop innovative high-efficiency energy storage systems.^1−6^ Supercapacitors have been identified as attractive options for energy storage due to their high power density, fast charge/discharge rate, and long cycle life.^7−11^ However, the poor energy density and low operating voltage of supercapacitors have hindered their widespread and practical implementation.^12^ Therefore, achieving high energy density while maintaining high power density is essential to meet the urgent demand for high-performance energy storage devices.

In recent years, various metal oxides with high theoretical specific capacitances have been widely investigated as electrode materials for supercapacitors.^13−16^ However, most of the metal oxides have poor electrical conductivity, resulting in poor rate and cycle performance in supercapacitors.^17,18^ Recently, certain transition metal chalcogenides have attracted much attention due to their higher electrical conductivity, reduced band gap, and excellent electrochemical activity compared to their transition metal oxide analogues.^19^ In particular, iron sulfides have attracted much interest due to their low cost, high theoretical capacity (890 mA h g^–1^), narrow band gap, nontoxic nature, and abundant natural supply.^20,21^ However, the rate and power capability of iron sulfide-based electrodes need further improvement for their effective utilization in electrochemical energy storage systems. FeS_2_-based electrodes undergo significant volume expansion during cycling processes, which limits their application in commercial supercapacitors.^22^

One of the efficient techniques to improve the conductivity of the transition metal compounds while buffering the aggregation and volume expansion during the charge/discharge processes is to composite them with highly conductive carbon materials such as carbon microspheres, carbon nanotubes (CNTs), and graphene.^23−26^ These carbon matrices can provide highly conductive channels for rapid charge transport to enhance the electrochemical reaction kinetics.^27^ Huang et al. prepared three-dimensional porous carbon (3DPC) decorated with FeS_2_ nanosphere nanocomposites (FeS_2_/3DPC) as electrode material for supercapacitors by a facile hydrothermal approach.^28^ The FeS_2_/3DPC nanocomposites exhibited much better electrochemical energy storage performance than pure FeS_2_. The FeS_2_/3DPC electrode exhibits a specific capacitance of 304 F g^–1^ at 2 A g^–1^. The use of 3DPC as FeS_2_ substrate can improve the conductivity of the electrode material and effectively inhibit the volume collapse of FeS_2_. Liu et al. reported a facile hydrothermal method coupled with an etching method to synthesize passion fruit-like FeS_2_@Carbon microspheres.^29^ The FeS_2_@Carbon microsphere-based electrode exhibits electrochemical performance with a specific capacity of 278.4 F g^–1^ at 1 A g^–1^ and a capacity retention of 57.7% at 5 A g^–1^ after 10,000 cycles. Sridhar et al. reported a simple one-pot, two-step technique for the synthesis of carbon nanofiber (CNF) cross-linked FeS_2_ networks by microwave pyrolysis of ferrocene to iron-decorated CNF and its subsequent sulfidation by a sustainable source.^22^ When applied as supercapacitor electrodes, the microwave-synthesized FeS_2_/CNF electrodes exhibit a high capacitance of 612 Fg^1–^ at 5 mVs^–1^ and with 97% capacitance retention after 2000 cycles. Balakrishnan et al. used a simple hydrothermal technique to prepare iron disulfide microspheres anchored on a reduced graphene oxide matrix (rGO-FeS_2_ hybrid).^30^ Due to its enhanced electrical conductivity and high surface area, the rGO-FeS_2_ hybrid microsphere electrode exhibited an enhanced areal capacitance of 112.41 mF cm^–2^ compared to the pure FeS_2_ microflower electrode (70.98 mF cm^–2^). A porous RGO/FeS composite grown on the Fe foil surface by a simple one-pot hydrothermal method exhibited a high specific capacitance of 900 mF cm^–2^ (300 F g^–1^). The Fe foil served multiple roles as Fe source, GO reducer, and subsequent current collector of the electrode. The presence of RGO effectively prevents the aggregation of FeS nanosheets. In addition, RGO acts as a buffer layer that reduces FeS volume change and maintains structural integrity during charging and discharging, resulting in superior cycling stability of 97.5% maximum capacity retention after 2000 cycles.^31^ Hassanpoor et al. reported a new simple chemical method to prepare the pyrite-sulfur-doped reduced graphene oxide nanocomposite (FeS_2_–SRGO) as the electrode of the supercapacitor.^32^ The FeS_2_–SRGO electrode exhibited a specific capacitance of 277 F g^–1^. The capacity retention is about 90% of the initial capacity after 200 charge–discharge cycles.

Among the various morphologies of FeS_2_ materials, such as nanorods, nanowires, nanocubes, nanoflakes, etc., spherical morphologies with hierarchically structured FeS_2_ are found to be more advantageous for achieving fast charge transfer and easy electrolyte diffusion properties.^20^ Despite the considerable progress in tailoring FeS_2_ structures, the performance of FeS_2_-based electrodes remains unsatisfactory. The potential windows of these iron sulfide-based electrodes in aqueous electrolytes have an operating potential window of about 1.0–1.3 V.^33^ In addition, the aforementioned FeS_2_ powder materials must be combined with insulating binders and conductive additives, which drastically reduce the advantages of nanoscale materials by adding unwanted interfaces that increase internal resistance.^34^ Therefore, the development of new methods to create unique FeS_2_-based nanocomposites to further improve energy storage performance is highly desirable but challenging. Free-standing FeS_2_ composite electrodes are being developed in parallel with research on nanoscale FeS_2_ powder materials because free-standing FeS_2_ composites could retain the advantages of short diffusion paths for ions/electrodes and larger surface area by avoiding the use of polymer binders and additives.

In this work, a regenerated cellulose (RC)/CNT film was prepared by introducing a CNT dispersion into the cellulose solution and vigorously stirring the mixture. After stirring for 1 h, the RC/CNT film was formed by vacuum suction. Subsequently, the RC/CNT composite film was immersed in a mixed solution containing urea, sodium thiosulfate pentahydrate (Na_2_S_2_O_3_·5H_2_O), and iron(III) chloride hexahydrate (FeCl_3_·6H_2_O). The reaction occurred in an oil bath at 100 °C for 5 h. Through this simple chemical bath deposition method, flexible RC/CNT/FeS_2_ composite films with varying FeS_2_ content were obtained. Furthermore, the RC/CNT/FeS_2_ composite film was soaked in an aqueous solution containing pyrrole. The film absorbed pyrrole, which was then immersed in an aqueous ferric chloride solution to initiate the polymerization of pyrrole. This process resulted in the fabrication of RC/CNT/FeS_2_/PPy composite films. A flexible RC matrix, allowed the homogeneous distribution of CNTs without severe agglomeration due to strong hydrogen bonding between them. A porous network structure with many open pores and channels was created by these interlaced cellulose fibers, providing a flexible cellulose film suitable as a substrate for the incorporation of electrochemically active materials to form functional composite materials for potentially many applications.^35,36^ The RC/CNT conductive film having large pores and high surface area was an excellent template for the incorporation of FeS_2_. In addition, studies have shown that the incorporation of conductive polymers such as PPy, polyaniline, and PEDOT can significantly improve the ion transport, electrical conductivity, and cycling stability of the transition metal compound-based electrodes.^37^ Due to its advantages such as easy synthesis, low cost, and excellent environmental stability, PPy was coated on the RC/CNT/FeS_2_ composite film to further improve its conductivity and electrochemical properties.^38^ Moreover, the application of a PPy coating would effectively inhibit the desorption of FeS_2_ from the composite film of RC/CNT. The unique microflower-like morphology of FeS_2_, the high conductivity and surface area provided by the RC/CNT substrate, and the excellent redox activity of PPy can be attributed to the remarkable electrochemical properties of the RC/CNT/FeS_2_/PPy composite electrodes. The chemical structure and morphology of the RC/CNT/FeS_2_/PPy composite films were characterized using various analytical techniques, including the Fourier transform infrared (FTIR) spectroscopy, X-ray photoelectron spectroscopy (XPS), scanning electron microscopy (SEM), high-resolution transmission electron microscopy (HRTEM), and X-ray diffractometer (XRD). Additionally, electrochemical characterization of the prepared RC/CNT/FeS_2_/PPy composite films was performed using an electrochemical workstation. This involved testing the electrochemical properties, such as capacitance, charge–discharge behavior, and cyclic stability, to evaluate the performance of the composite films as electrodes for supercapacitors. The RC/CNT/FeS_2_/PPy electrode prepared under optimal conditions was used to fabricate a symmetric supercapacitor. The device could achieve a wide voltage window of 1.4 V. Moreover, it also provided a high areal capacitance of 1280 mF cm^–2^, a maximum energy density of 329 μWh cm^–2^, a maximum power density of 24.9 mW cm^–2^, and 86.2% of capacitance retention after 10,000 cycles at 40 mA cm^–2^.

## 2 Experimental Section

### 2.1 Materials

Kapok fiber (KF, *Ceiba pentandra*) and CNTs were procured from Unionward, Taiwan, and C-nano Technology, China, respectively. Nitric acid (HNO_3_), sulfuric acid (H_2_SO_4_), urea [CO(NH_2_)_2_], ferric chloride hexahydrate (FeCl_3_·6H_2_O), and ferrous chloride tetrahydrate (FeCl_2_·4H_2_O) were purchased from J. T. Baker Chemicals. Thiourea [SC(NH_2_)_2_], sodium hydroxide (NaOH), sodium thiosulfate (Na_2_S_2_O_3_·5H_2_O), and sodium sulfate (Na_2_SO_4_) were procured from Honeywell Fluka. Acetic acid (AcOH) and sodium chlorite (NaClO_2_) were purchased from Sigma–Aldrich.

### 2.2 RC/CNT Composite Film

The untreated CNTs were added to 3 M HNO_3_ and refluxed at 90 °C for 14 h. The treated CNTs were then ultrasonicated for 1 h, rinsed with DI water to neutral pH, filtered, and dried under vacuum at 80 °C for 12 h to obtain oxidized CNTs.^39^ These oxidized CNTs were used in this study. Uniform dispersion of oxidized CNTs in DI water was prepared by ultrasonication. Cellulose was extracted from KF using an AcOH solution containing NaClO_2_.^35^ The extracted cellulose was then dissolved in NaOH/urea/thiourea aqueous solution. The CNT dispersion was added dropwise to the cellulose solution under vigorous magnetic stirring. After stirring for 1 h, the RC/CNT film was formed by vacuum suction. The mass ratio of regenerated cellulose to treated CNT was kept constant at 3:7.

### 2.3 RC/CNT/FeS_2_ Composite Films

**Figure 1:**
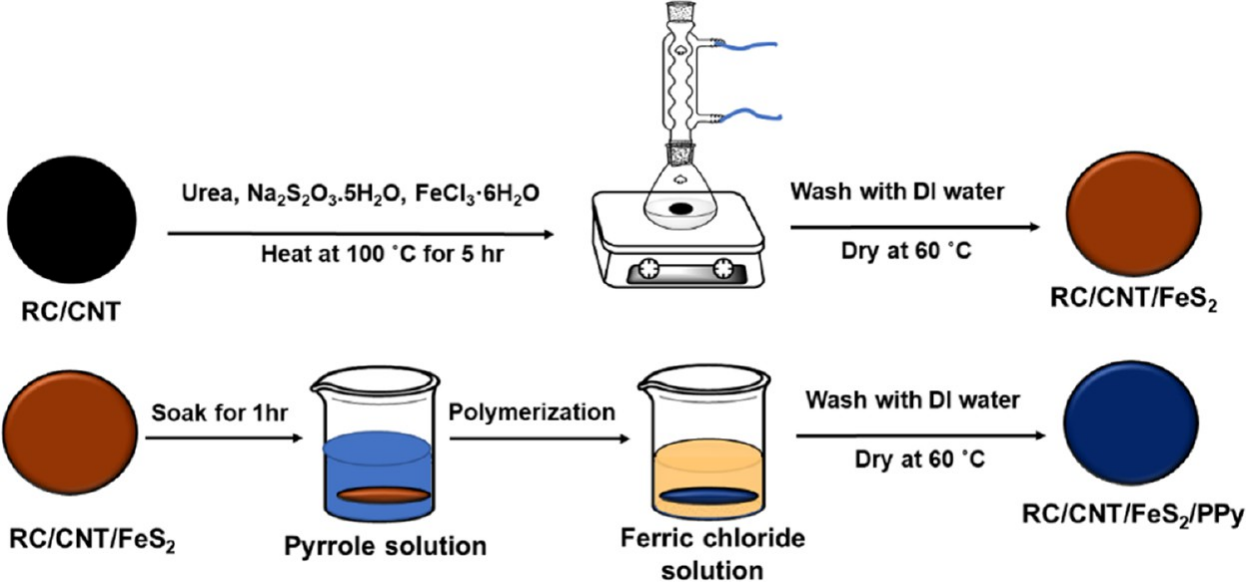
Schematic illustration of the RC/CNT/FeS_2_/PPy and RC/CNT/FeS_2_/PPy composite film synthesis processes.

As shown in Figure 1, urea (1.68 g), Na_2_S_2_O_3_·5H_2_O (4.42 g), and FeCl_3_·6H_2_O were dissolved in 70 mL of DI water under magnetic stirring at room temperature. The RC/CNT composite film was immersed in the above solution and reacted in an oil bath at 100 °C for 5 h. After the reaction, the composite film was thoroughly washed with DI water and dried at 60 °C. The RC/CNT/FeS_2_ composites prepared with different ferric chloride concentrations of 0.1, 0.2, 0.3, and 0.5 M were named RC/CNT/0.1FeS_2_, RC/CNT/0.2FeS_2_, RC/CNT/0.3FeS_2_, and RC/CNT/0.5FeS_2_, respectively. The amount of FeS_2_ deposited on the RC/CNT film was calculated by measuring the weight of the RC/CNT film before and after FeS_2_ deposition.

### 2.4 RC/CNT/FeS_2_/PPy Composite Films

As shown in Figure 1, the RC/CNT/FeS_2_ composite film was soaked in an aqueous solution (20 mL) containing 1 mL of pyrrole for 1 h. The film with absorbed pyrrole was then placed in an aqueous ferric chloride solution to initiate the polymerization of pyrrole. After the polymerization reaction was completed, the composite films were washed with DI water and then dried at 60 °C for 12 h in a vacuum oven. The RC/CNT/FeS_2_ composite films prepared at different polymerization times of 15, 30, 60, and 75 min were named RC/CNT/0.3FeS_2_/PPy-15, RC/CNT/0.3FeS_2_/PPy-30, RC/CNT/0.3FeS_2_/PPy-60, and RC/CNT/0.3FeS_2_/PPy-75, respectively.

### 2.5 Materials Characterization

A PerkinElmer RX1 infrared spectrophotometer was used to obtain the FTIR spectra of the samples. The surface components of the samples were analyzed by XPS (ESCALAB 250Xi, Thermo Fisher). Dynamic light scattering spectroscopy (Litesizer DLS 500, Anton Paar) was used to measure the ζ-potentials of cellulose, CNT, FeS_2_, and PPy. The energy band gaps and highest occupied molecular orbital (HOMO) energy levels of CNT, FeS_2_, and PPy were determined through the utilization of ultraviolet–visible (UV–vis) absorption (Hitachi U3010 UV–vis spectrometer) and photoelectron spectroscopies (Riken Keiki surface analyzer model AC-2). The XRD patterns of the powder samples were identified by using a Bruker D8 ADVANCE diffractometer equipped with a NaI scintillation counter and using monochromatized Cu-Kα radiation (λ = 1.5406 Å). The measurements of XRD were performed in the range of 2θ = 10–80° with an increment of 2°/min. Thermogravimetric analysis (TGA) was performed using a PerkinElmer Pyris1 thermogravimetric analyzer (N_2_ atmosphere, 10 °C min^–1^ heating rate). Morphologies of the composite films were analyzed using SEM (Jeol-JSM-7401F, operating voltage: 3 kV, Japan) and HRTEM (JEOL JEM-1400). Prior to analysis, the samples were mounted on microscope stubs using a double-sided conductive tape and coated with a thin layer of evaporated platinum. A drop of the sample dispersed in ethanol was placed on a Cu grid to collect the TEM images of dried samples using a transmission electron microscope (TEM, JEOL JEM-2100, Japan) operated at an accelerating voltage of 200 kV. The surface areas and pore characteristics were determined using a surface area and porosity analyzer (ASAP 2010, Micromeritics).

### 2.6 Electrochemical Measurement

Electrochemical characterization (cyclic voltammetry, CV; galvanostatic charge/discharge, GCD; electrochemical impedance spectroscopy, EIS) of the prepared composites was performed using an electrochemical workstation (CH Instruments). A three-electrode system consisting of a piece of the composite film, a platinum foil, and a saturated calomel electrode (SCE) as the working, counter, and reference electrodes, respectively, was used to investigate the electrochemical performance of the composite film in 1 M Na_2_SO_4_ aqueous electrolyte. The following eqs 1 and 2 were used to calculate the areal capacitance (*C*_A_, mF cm^–2^) and gravimetric capacitance (*C*_m_, F g^–1^) of the composite electrodes:^40^
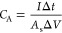
1
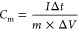
2where *I*, Δ*t, A*_s_, *m*, and Δ*V* denote the discharge current (A), discharge time (s), area of the freestanding electrode (cm^2^), weight of the freestanding electrode (g), and the potential window (V), respectively.

### 2.7 Symmetric Supercapacitor

The symmetric supercapacitor device was fabricated by sandwiching a filter paper separator soaked in 1 M Na_2_SO_4_ electrolyte between two identical pieces of RC/CNT/0.3FeS_2_/PPy-60 composite films. Nickel foam was used as the current collector. Equations 3–5 were used to calculate the areal capacitance (*C*_cell_, F cm^–2^), energy density (*E*, μW h cm^–2^), and power density (*P*, μW cm^–2^) of the fabricated SC, respectively.^39^
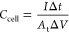
3
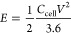
4
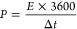
5where *I*, Δ*t*, Δ*V*, and *A*_t_ denote the discharge current (A), discharge time (s), the potential window (V), and geometric electrode working area (cm^2^), respectively.

## 3 Results and Discussion

### 3.1 Chemical Structures Characterization

For the deposition of FeS_2_ on RC/CNT, experiments were carried out using different ratios of FeCl_3_ and Na_2_S_2_O_3_. The chemical structures of RC/CNT/FeS_2_ and RC/CNT/FeS_2_/PPy nanocomposites were confirmed by FTIR spectra. Figure S1 shows the FTIR spectra of the RC, CNT, RC/CNT, RC/CNT/0.3FeS_2_, and RC/CNT/0.3FeS_2_/PPy-60 films. The characteristic vibrational bands of the RC appeared at 3415, 2914, 1644, 1430, 1369, 1070, and 849 cm^–1^ were attributed to the stretching of O–H groups, the asymmetric stretching of aliphatic C–H units in pyranose rings, C=O bonds of carboxylic acid groups, H–O–H bending of absorbed water, symmetric bending of −CH_2_ units of pyranose rings, O–H bending, C–O–C pyranose ring skeleton vibrations, and C1–H deformation vibrations, respectively.^41^ After the introduction of CNTs into the RC matrix, the FTIR spectra of the RC/CNT (3:7, w/w) retained the typical characteristic peaks of CNT, which was attributed to the high content of CNT in the RC/CNT film. The absorption peaks at 1700, 1533, and 1050 cm^–1^ were assigned to the stretching modes of C=O, C=C, and C–O from carboxylic acid groups on CNT. For the RC/CNT/0.3FeS_2_ samples, the stretching absorptions of Fe=S and Fe–S were observed at 1050–1156 and 607–625 cm^–1^, respectively.^42^ The absorption intensity of FeS_2_ was increased with increasing FeS_2_ content for the RC/CNT/FeS_2_ samples. In addition, the spectrum of the RC/CNT/0.3FeS_2_/PPy-60 nanocomposite showed the characteristic absorption bands of PPy, but those of RC, CNT, and FeS_2_ had disappeared, indicating that the RC/CNT/0.3FeS_2_ was almost completely covered by PPy. The spectrum of PPy coated on RC/CNT/0.3FeS_2_ shows a series of broad absorptions for different stretching modes at 1539 (C=C), 1456 (C–N), 1158 (C–N), and 892 (N–H wagging of the PPy ring) cm^–1^, and different characteristic C–H bending modes (747, 1059 cm^–1^) for the RC/CNT/0.3FeS_2_/PPy60 nanocomposites.^43,44^

XPS analysis revealed the compositions and elemental states of Fe, S, and O atoms in the RC/CNT/0.3FeS_2_ composite film (Figure S2). The survey spectrum (Figure S2(a)) showed the presence of Fe, S, O, and C elements in the composite film. As shown in Figure S2(b), two peaks in the high-resolution Fe 2p spectrum located at 711.2 and 724.5 eV could be attributed to Fe 2p3/2 and Fe 2p1/2, respectively.^20^ Furthermore, the peak at 719.4 eV could be assigned to the satellite peaks corresponding to the oxidation of FeS_2_. In Figure S2(c), the S 2p spectrum could be divided into two peaks, and the peaks at 163.7 and 164.8 eV belong to S 2p3/2 and S 2p1/2, respectively.^21^ In Figure S2(d), the peak at 532.2 eV for the O 1s orbital was attributed to the C–O bonds of CNT and RC.^12^ Therefore, the XPS analysis showed that the FeS_2_ on the RC/CNT composite film was well prepared for the RC/CNT/0.3FeS_2_ composite film. Similar XPS spectra were observed for the RC/CNT/0.1 FeS_2_ and RC/CNT/0.2 FeS_2_ composite films.

The ζ-potential analysis was employed to determine the surface charges of cellulose, CNT, FeS_2_, and Ppy. ζ-potentials of −27.5, 25.9, −10.3, and 29.5 mV were determined for cellulose, CNT, FeS_2_, and PPy, respectively. This result suggests that positively charged CNT will interact with negatively charged cellulose. Adherence of the negatively charged FeS_2_ particles to the positively charged CNT fibers is preferred. Furthermore, it can be observed that the positively charged PPy particles would demonstrate favorable compatibility with the negatively charged FeS_2_ particles. Charge transfer would be facilitated by robust interactions between the RC/CNT, FeS_2_, and PPy layers.^45^

Figure S3 illustrates the tauc-plots, AC-2 low-energy photoelectron spectra, and energy levels of the CNT, FeS_2_, and PPy materials. The CNT, FeS_2_, and PPy materials exhibited HOMO energy levels of −5.02, −5.22, and −4.80 eV, respectively, in conjunction with energy band gaps of 2.75, 3.82, and 3.77 eV. Hence, −2.27, −1.40, and −1.03 eV were the minimum unoccupied molecular orbital (LUMO) energies of the CNT, FeS_2_, and PPy substances, respectively. During the redox reaction, the elevated LUMO values of PPy and FeS_2_ facilitate charge transfer from the PPy/FeS_2_ layer to the RC/CNT layer and the current collector.

### 3.2 Morphology of the RC/CNT/FeS_2_/PPy Composite Films

The XRD patterns of the RC, CNT, RC/CNT, and RC/CNT/0.3FeS_2_ composite films are shown in Figure S4. The XRD pattern of RC showed distinct diffraction peaks at 2θ values of 12, 20, 22, and 34.6°, representing the (1i̅0), (110), (020), and (004) crystalline planes of cellulose II, respectively.^46,47^ The diffraction pattern of RC/CNT showed diffraction peaks at 2θ values of 25.6 and 43°, representing the (002) and (101) interplanar spacings of the CNTs, respectively, confirming the incorporation of the CNTs on the cellulose matrix.^35^ The XRD pattern of the RC/CNT/0.3FeS_2_ composite showed peaks at values of 2θ of 33.2, 36.7, 41.1, 47.8, 55.8, 58.9, 61.3, 63.8, and 76.4° correspond to (200), (210), (211), (220), (311), (222), (023), (321), and (331) planes, respectively, of pyrite FeS_2_ (JCPDS card no:00–042–1340).^21^ In addition, the crystalline peaks of cellulose and CNTs were absent in the XRD pattern of the RC/CNT/0.3FeS_2_ composite, indicating the complete coverage of FeS_2_ on the surface of RC/CNT. Similar results were observed for the RC/CNT/0.1FeS_2_ and RC/CNT/0.2FeS_2_ composite films. In addition, the XRD patterns of the RC/CNT/PPy-60, RC/CNT/0.3FeS_2_/PPy-15, RC/CNT/0.3FeS_2_/PPy-30, RC/CNT/0.3FeS_2_/PPy-60, and RC/CNT/0.3FeS_2_/PPy-75 composite films are shown in Figure S5. For these PPy deposited RC/CNT/0.3FeS_2_/PPy films, in addition to the crystalline peaks of FeS_2_, a broad peak centered at about 22° attributed to the amorphous PPy. The presence of a high deposition content of PPy in the RC/CNT/0.3FeS_2_/PPy-75 composite film leads to the absence of diffraction peaks associated with FeS_2_.

**Figure 2:**
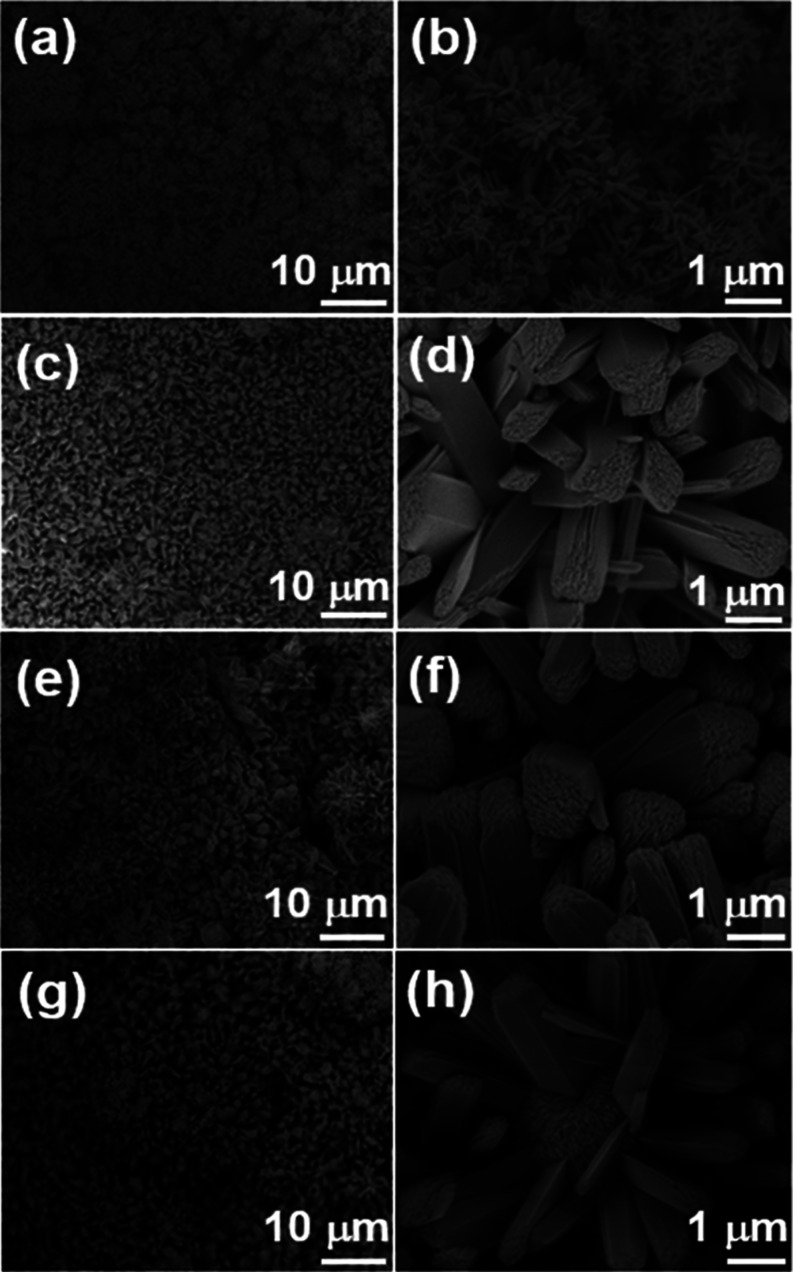
SEM images of (a, b) RC/CNT/0.1FeS_2_, (c, d) RC/CNT/0.2FeS_2_, (e, f) RC/CNT/0.3FeS_2_, and (g, h) RC/CNT/0.5FeS_2_ composite films.

**Figure 3:**
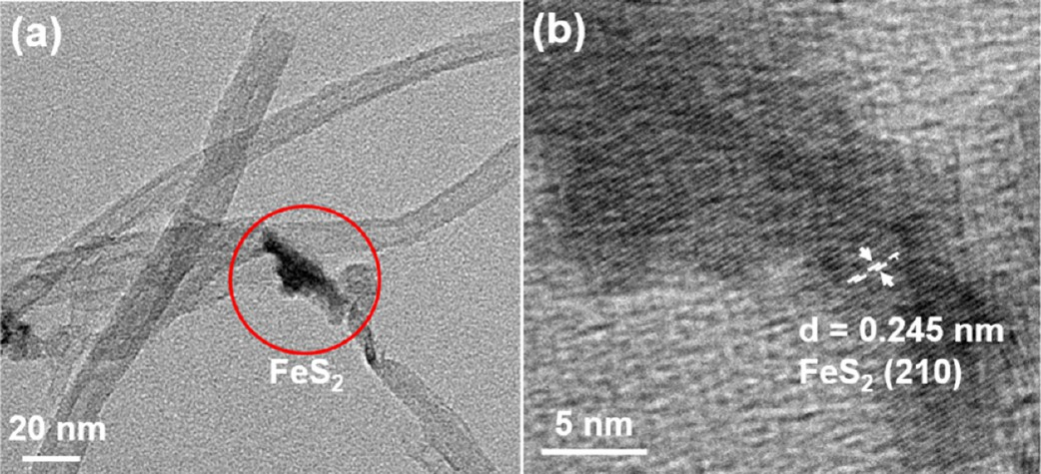
HRTEM images of (a) FeS_2_/CNT and (b) FeS_2_ samples.

**Figure 4:**
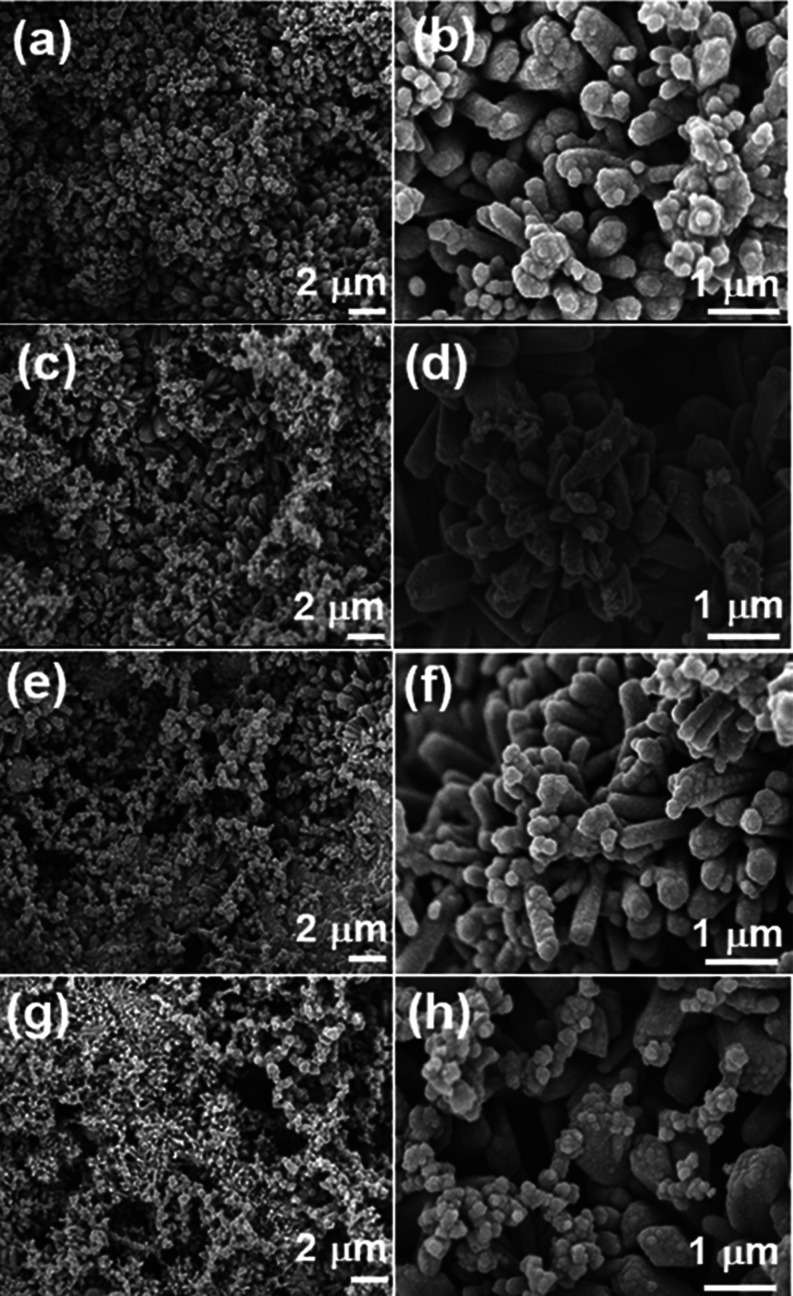
SEM micrographs of (a, b) RC/CNT/0.3FeS_2_/PPy-15, (c, d) RC/CNT/0.3FeS_2_/PPy-30, (e, f) RC/CNT/0.3FeS_2_/PPy-60, and (g, h) RC/CNT/0.3FeS_2_/PPy-75 composite films.

**Figure 5:**
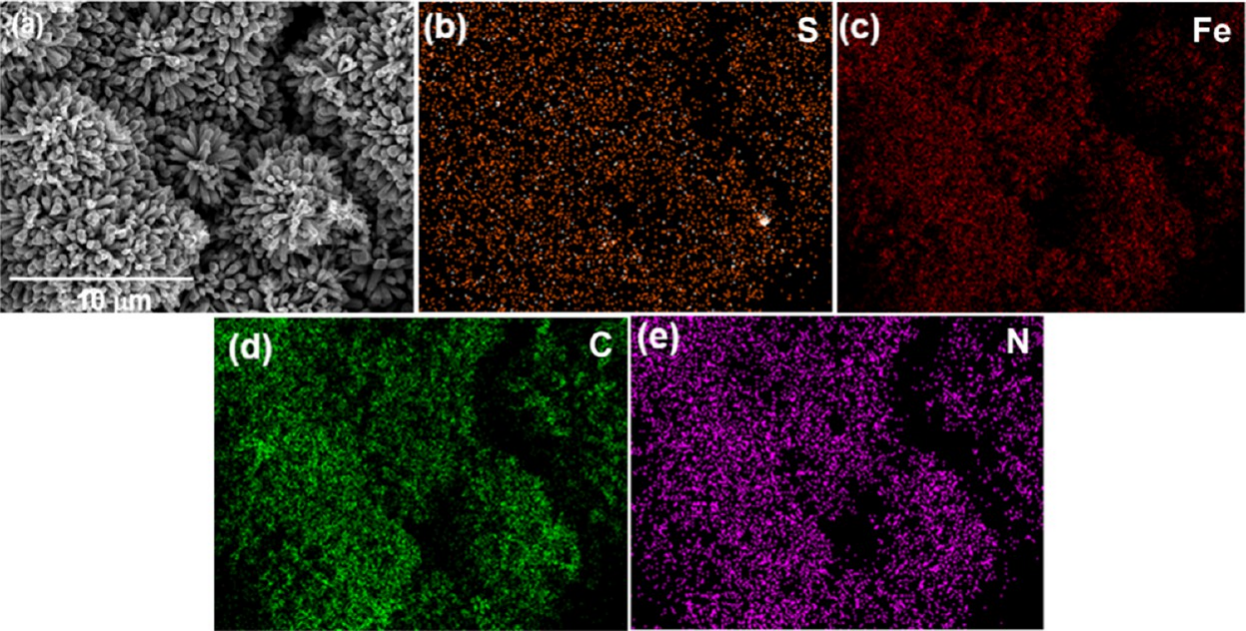
(a) SEM image and (b–e) EDX images of (b) S, (c) Fe, (d) C, and (e) N in the RC/CNT/0.3FeS_2_/PPy-60 composite.

SEM was used to observe the surface morphologies of the composite samples. The RC fibers formed by dissolving KF in cellulose solvent and then regenerating in DI water showed a porous fibrous structure with a rough, wrinkled surface (Figure S6(a)). The SEM image of the RC/CNT showed a dense coating of randomly entangled CNTs on the surface of RC fibers (Figure S6(b)). The high porosity and conductivity of the RC/CNT film suggest that it would be an excellent substrate for incorporating electrochemically active materials. After the chemical deposition process, FeS_2_ microflowers anchored on the RC/CNT matrix can be seen in the SEM images of the RC/CNT/0.1FeS_2_, RC/CNT/0.2FeS_2_, RC/CNT/0.3FeS_2_, and RC/CNT/0.5FeS_2_ composite films (Figure 2). Meanwhile, the distribution of FeS_2_ on the surface of the RC/CNT composite film was characterized by the energy-dispersive X-ray (EDX) images (Figure S7). Mapping analysis clearly identifies the S and Fe element distributions corresponding to the SEM image of the RC/CNT/0.3FeS_2_. High concentration of S and Fe elements was observed for the RC/CNT/0.3FeS_2_ electrode. The EDX images confirmed that the FeS_2_ nanoparticles were uniformly distributed on the surface of the RC/CNT composite film. Furthermore, a high-resolution TEM (HRTEM) image of the FeS_2_ deposited on the CNT is presented in Figure 3. This image illustrates lattice fringes spaced at 0.245 nm apart, which correspond to the (210) facet of the cubic FeS_2_. FeS_2_ has a (210) interplanar spacing of 0.24 nm.^48^ After PPy deposition, the PPy deposited on the rods of FeS_2_ microflowers is visible in the SEM images of the RC/CNT/0.3FeS_2_/PPy-15, RC/CNT/0.3FeS_2_/PPy-30, RC/CNT/0.3FeS_2_/PPy-60, and RC/CNT/0.3FeS_2_/PPy-75 composite films (Figure 4). The PPy particles deposited on the surface of FeS_2_ rods and the interstitial spaces can be seen in the SEM images of RC/CNT/0.3FeS_2_/PPy composites. The EDX images obtained for the RC/CNT/0.3FeS_2_/PPy-60 composite provided evidence supporting the uniform distribution of PPy particles across the surface of the RC/CNT/0.3FeS_2_ composite film (Figure 5). Furthermore, the composite material RC/CNT/0.3FeS_2_/PPy-75 exhibited the phenomenon of PPy nanoparticle aggregation on the surface of FeS_2_ (Figure 4g,h).

To gain a deeper understanding of the thermal properties of the materials, TGA was used to record the weight loss in percent with increasing temperature of the composites in a nitrogen atmosphere (Figure S8). The weight loss below 200 °C for all samples can be attributed to the loss of absorbed water. In Figure S8(a), the TGA curve of RC showed a significant weight loss from 250 to 400 °C due to thermal degradation of the crystalline regions of cellulose and degradation of glucosidic units.^36^ The inclusion of thermostable CNTs is responsible for the higher char production of RC/CNT composite compared to RC. Pure FeS_2_ exhibits two major stages of weight loss. The first stage of weight loss occurs between 200 and 280 °C and is mainly due to absorbed and intermolecular water. The second stage of weight loss occurs between 450 and 500 °C. At this stage, weight loss is caused by the thermal decomposition of iron disulfide. The thermal decomposition of FeS_2_ produces pyrrhotite (Fe_1–*x*_S) and S_2_ gas. The thermal decomposition reaction of FeS_2_ in a nitrogen atmosphere can be written as (1 – *x*)FeS_2_ ↔ Fe_(1–*x*)_ S + (0.5 – *x*)S_2_(g).^49,50^ As the iron precursor concentration increases, more iron sulfide is deposited on the RC/CNT composite, resulting in the release of more S_2_ gas during thermal decomposition. Therefore, the weight loss increases with increasing FeS_2_ content and the final residual weight of RC/CNT/FeS_2_ composites decreases. The residual weights of RC/CNT/0.1FeS_2_, RC/CNT/0.2FeS_2_, RC/CNT/0.3FeS_2_, and RC/CNT/0.5FeS_2_ were 73.9, 70.7, 58, and 44%, respectively. For the RC/CNT/FeS_2_/PPy composites, the first weight loss stage of thermal decomposition became smoother, and a gradual weight loss pattern was observed after the deposition of PPy, as shown in Figure S8(b). The residual weights of RC/CNT/0.3FeS_2_/PPy-15, RC/CNT/0.3FeS_2_/PPy-30, RC/CNT/0.3FeS_2_/PPy-60, and RC/CNT/0.3FeS_2_/PPy-75 composites were 68.2, 70.1, 72.0, 72.0, and 73.1%, respectively. The higher char yield with increasing PPy deposition indicates the higher thermal stability of RC/CNT/FeS_2_/PPy composites compared to RC/CNT/0.3FeS_2_ composites.

Porous features of supercapacitor electrode materials have been shown to significantly affect electrolyte transport and ion diffusion properties.^51^ The N_2_ adsorption and desorption isotherms and BJH pore size distribution of RC/CNT, RC/CNT/FeS_2_, and RC/CNT/FeS_2_/PPy composites are shown in Figures S9 and 6, respectively. The Brunauer–Emmett–Teller (BET) surface areas of RC/CNT, RC/CNT/0.1FeS_2_, RC/CNT/0.2FeS_2_, RC/CNT/0.3FeS_2_, RC/CNT/0.5FeS_2_, RC/CNT/0.3FeS_2_/PPy-15, RC/CNT/0.3FeS_2_/PPy-30, RC/CNT/0.3FeS_2_/PPy-60, and RC/CNT/0.3FeS_2_/PPy-75 composites were 128.6, 95.3, 70.8, 53.3, 47.5, 54.2, 54.8, 72.5, and 60.5 m^–2^g^–1^, respectively. The corresponding mean pore diameters were 21.3, 20.3, 21.2, 21.7, 21.5, 18.8, 20.2, 22.2, and 22.5 nm, respectively. The decrease in surface areas with increasing FeS_2_ content for RC/CNT/FeS_2_ composites is probably due to possible pore blockage as the iron sulfide content was increased. However, the surface areas increased for the RC/CNT/FeS_2_/PPy composites as a result of PPy deposition. The conversion of rod-shaped FeS_2_ into hollow spheres can be attributed to the increased surface area after PPy deposition. The hysteresis loops in the relative pressure range of 0.8–1.0 in the adsorption–desorption isotherms showed a type IV isotherm, indicating that all the materials were mesoporous. The BJH pore size distribution curves confirmed that the main pore size distribution of the samples was in the mesoporous region. The mesoporous structure of the composite materials helps to increase the electrode–electrolyte contact areas, resulting in improved charge storage performance.

**Figure 6:**
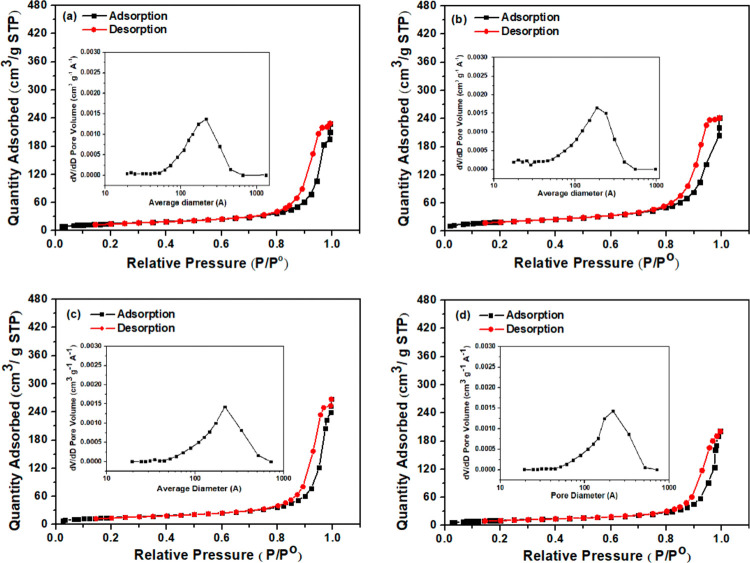
N_2_ adsorption–desorption isotherms and pore size distributions of (a) RC/CNT/0.3FeS_2_/PPy-15, (b) RC/CNT/0.3FeS_2_/PPy-30, (c) RC/CNT/0.3FeS_2_/PPy-60, and (d) RC/CNT/0.3FeS_2_/PPy-75 composites films.

### 3.3 Electrochemical Properties of the RC/CNT/FeS_2_/PPy Composite Films-Based Electrodes

**Figure 7:**
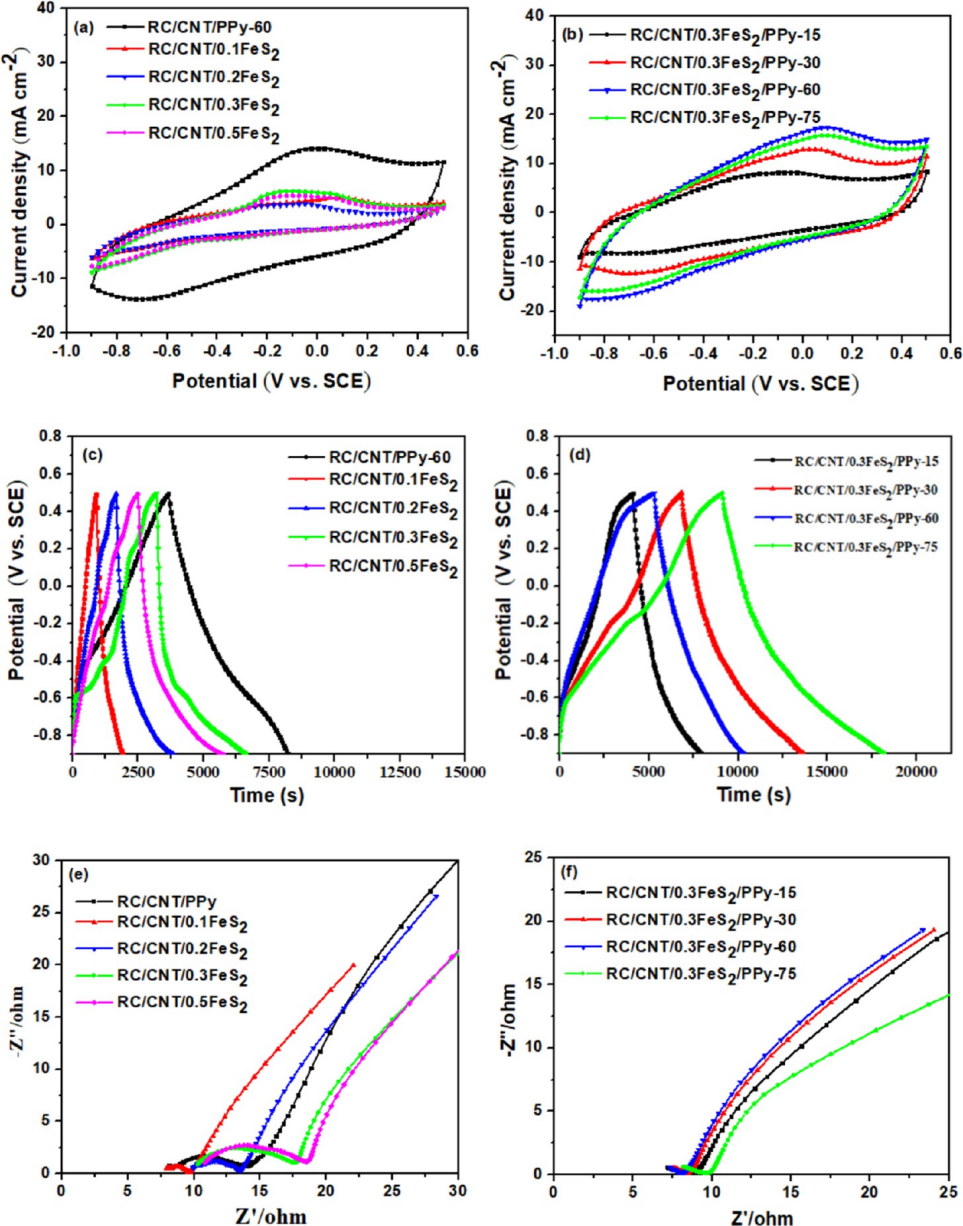
Electrochemical performances of the RC/CNT/0.1FeS_2_, RC/CNT/0.2FeS_2_, RC/CNT/0.3FeS_2_, RC/CNT/0.5FeS_2_, RC/CNT/0.3FeS_2_/PPy-15, RC/CNT/0.3FeS_2_/PPy-30, RC/CNT/0.3FeS_2_/PPy-60, RC/CNT/0.3FeS_2_/PPy-75, and RC/CNT/PPy-60 composites ((a, b) CV plots (scan rate: 5 mV s^–1^), (c, d) GCD plots (current density: 1 mA cm^–2^), and (e, f) EIS).

The electrochemical properties of the composite electrodes were evaluated in a potential range of −0.9–0.5 V in a three-electrode setup. The CV curves of the electrodes at 5 mV s^–1^ are shown in Figure 7a. The capacitance of the electrodes was derived from a contribution of the EDLC of the CNTs and the pseudocapacitance of the FeS_2_ and PPy, as indicated by the slightly distorted rectangular-shaped CV curves. The composites of RC/CNT/FeS_2_ exhibited slightly distorted CV curves as a result of the pseudocapacitance generated by the rapid and reversible charge-transfer reactions occurring on the surface of FeS_2_. As shown in eq 6, the electrochemical interaction of Fe atoms with the electrolyte ions leads to the pseudocapacitance behavior.^30^
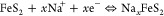
6where *x* is the mole fraction of inserted Na^+^ ions.

Since the capacitance characteristic is proportional to the area enclosed by the CV curve, the areas of the CV curves for the RC/CNT/FeS_2_/PPy composite electrodes exhibited significant enlargement compared to the RC/CNT/FeS_2_ composite electrodes, as depicted in Figure 7a,b. Significant redox peaks were observed in the CV curves of the composite electrodes consisting of RC/CNT/FeS_2_/PPy. The RC/CNT/0.3FeS_2_/PPy-60 composite electrode with the largest enclosed CV area provides the best capacitance characteristic among all the electrodes. The higher capacitance of RC/CNT/FeS_2_/PPy electrodes compared to RC/CNT/FeS_2_ electrodes revealed the crucial role of PPy in the enhanced electrochemical performance of the RC/CNT/FeS_2_/PPy electrodes.

The GCD plots of the composite electrodes measured at 1 mA cm^–2^ are shown in Figure 7c,d. The RC/CNT/FeS_2_/PPy electrodes had a significantly longer discharge time than the RC/CNT/FeS_2_ and RC/CNT/PPy electrodes, indicating a significantly higher specific capacitance, consistent with the CV results, due to the synergistic effects of the conductive CNTs and the well-distributed pseudocapacitive FeS_2_ and PPy. The areal capacitances of RC/CNT/0.1FeS_2_, RC/CNT/0.2FeS_2_, RC/CNT/0.3FeS_2_, RC/CNT/0.5FeS_2_, RC/CNT/0.3FeS_2_/PPy-15, RC/CNT/0.3FeS_2_/PPy-30, RC/CNT/0.3FeS_2_/PPy-60, RC/CNT/0.3FeS_2_/PPy-75, and RC/CNT/PPy-60 composites were 725.6, 1576.3, 2486.7, 2158.5, 2770.1, 3660.1, 6543.8, 5834.1, and 3229.83 mF cm^–2^, respectively, based on their GCD plots measured at 1 mA cm^–2^. The RC/CNT/0.3FeS_2_ composite exhibited a greater capacitance in comparison to the RC/CNT/0.1FeS_2_, RC/CNT/0.2FeS_2_, and RC/CNT/0.5FeS_2_ composites. According to the BET analysis, the surface areas of the RC/CNT/FeS_2_ composites decreased as the FeS_2_ content increased; this is likely due to the possibility of pore blockage caused by the increased iron sulfide content. For the RC/CNT/0.3FeS_2_ composite film, the optimal amount of FeS_2_ on the surface was observed. Furthermore, it was observed that the capacitances of the RC/CNT/0.3FeS_2_/PPy-30, RC/CNT/0.3FeS_2_/PPy-60, and RC/CNT/0.3FeS_2_/PPy-75 composite electrodes exhibited greater values in comparison to the RC/CNT/FeS_2_ and RC/CNT/PPy-60 composite electrodes. The electrochemical performance of the RC/CNT/FeS_2_/PPy composite electrode was found to be excellent, which can be attributed to the synergistic effect between FeS_2_ and PPy. As the PPy content increased, the electrochemical properties of the RC/CNT/0.3FeS_2_/PPy electrodes also improved. The astounding electrochemical properties of the RC/CNT/0.3FeS_2_/PPy-60 composite are attributed to its increased electroactive sites, enlarged surface area, fast electron and ion transport channel, and especially its easy electrolyte penetration. The RC/CNT/0.3FeS_2_/PPy-75 composite demonstrates a decrease in capacitance compared to the RC/CNT/0.3FeS_2_/PPy-60 composite. This can be attributed to the higher deposition content and aggregation of PPy on the FeS_2_ surface, as depicted in Figure 5g,h. The aggregation of PPy on the FeS_2_ surface leads to a reduction in the BET surface areas of the RC/CNT/0.3FeS_2_/PPy-75 composite. Consequently, there was insufficient interaction between the electrolyte and FeS_2_. Therefore, the RC/CNT/0.3FeS_2_/PPy-75 sample exhibited a lower capacitance value.

EIS is one of the essential factors to be examined when evaluating the internal resistance and capacitive behavior of supercapacitors. Figure 7e,f shows the Nyquist plots of RC/CNT/0.1FeS_2_, RC/CNT/0.2FeS_2_, RC/CNT/0.3FeS_2_, RC/CNT/0.5FeS_2_, RC/CNT/0.3FeS_2_/PPy-15, RC/CNT/0.3FeS_2_/PPy-30, RC/CNT/0.3FeS_2_/PPy-60, and RC/CNT/PPy-60 composites. The Nyquist plots showed a semicircle in the high-frequency region and an oblique line in the low-frequency region, corresponding to the charge transfer resistance (*R*_ct_) and Warburg diffusion impedance, respectively.^40^ The X-intercepts of the Nyquist plots correlate with the series resistance (*R*_s_). The deposition of FeS_2_ has shown a profound effect on the electrical conductivity and electrolyte diffusion properties of the RC/CNT/FeS_2_ nanocomposites. With the increase in FeS_2_ content, the *R*_ct_ and *R*_s_ values of the composites were increased, as shown in the inset of Figure 7e. Thus, the RC/CNT/0.5FeS_2_ electrode, which had the highest mass loading of FeS_2_, had higher *R*_s_ and *R*_ct_ values compared to our other composite electrodes. After the deposition of PPy, the *R*_s_ and *R*_ct_ values were significantly decreased due to the high conductivity of PPy compared to FeS_2_ (Figure 7f). However, the RC/CNT/PPy-60 composite showed much higher charge transfer resistance and diffusion resistance than the RC/CNT/FeS_2_/PPy composite electrodes. This result indicates that the synergistic combination of FeS_2_ and PPy efficiently reduced the *R*_s_ and *R*_ct_ values of the RC/CNT/FeS_2_/PPy composite electrodes.

**Figure 8:**
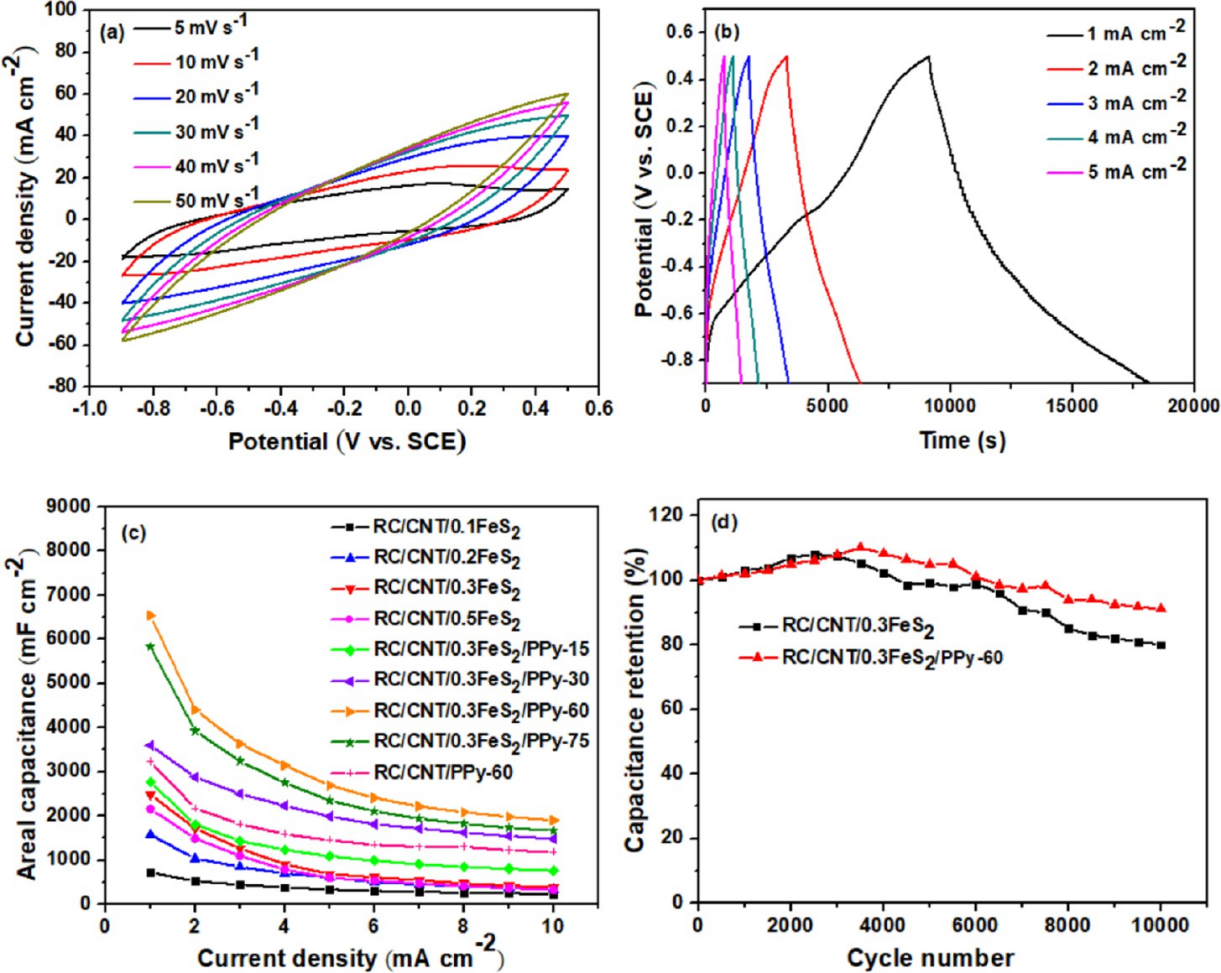
(a) CV plots at different scan rates and (b) GCD plots at different current densities of the RC/CNT/0.3FeS_2_/PPy-60 composite electrode. (c) Areal capacitances of the RC/CNT/0.1FeS_2_, RC/CNT/0.2FeS_2_, RC/CNT/0.3FeS_2_, RC/CNT/0.5FeS_2_, RC/CNT/0.3FeS_2_/PPy-15, RC/CNT/0.3FeS_2_/PPy-30, RC/CNT/0.3FeS_2_/PPy-60, RC/CNT/0.3FeS_2_/PPy-75, and RC/CNT/PPy-60 composites plotted with respect to the current density. (d) Cycling performance of the RC/CNT/0.3FeS_2_ and RC/CNT/0.3FeS_2_/PPy-60 electrodes, recorded at 15 mA cm^–2^.

Figure 8a shows CV plots of RC/CNT/0.3FeS_2_/PPy-60 composite electrode. The GCD plots of the RC/CNT/0.3FeS_2_/PPy-60 composite electrode are shown in Figure 8b. The GCD plots of the RC/CNT/0.3FeS_2_/PPy-60 electrode exhibited symmetrical triangular shapes at all current densities, revealing its superior capacitive properties. The areal and specific capacitances of the composite electrodes were calculated from the GCD plots; the results are summarized in Figures 8c and S10. Due to the poor utilization of the internal redox sites of the active materials at high discharge current densities, the areal and specific capacitances of all electrodes decreased with increasing current densities. In contrast, the electrodes composed of RC/CNT/FeS_2_/PPy exhibited greater areal and specific capacitances than those formed of RC/CNT/FeS_2_ and RC/CNT/PPy-60. The RC/CNT/0.3FeS_2_/PPy-60 electrode maintained a larger areal capacitance than the other composite electrodes at all current densities, demonstrating its superior rate performance. The synergistic effects of the higher active surface area of CNTs and the redox-active property of FeS_2_ microflowers and PPy may account for the superior performance of the RC/CNT/0.3FeS_2_/PPy-60 electrode. The FeS_2_ microflowers provide an open porous structure that allows electrolyte ions to easily reach the entire surface of the active materials, resulting in maximum utilization of the active materials. In addition, Figure S11 illustrates the relationship between areal capacitance values and the weight of the RC/CNT/FeS_2_ and RC/CNT/FeS_2_/PPy electrodes. The results indicate that the areal capacitance increases with a larger amount of FeS_2_ and PPy. However, an excessive amount of FeS_2_ and PPy leads to a decrease in the areal capacitance for both the RC/CNT/FeS_2_ and RC/CNT/FeS_2_/PPy electrodes. Since cycling stability is an important criterion for electrode materials, RC/CNT/0.3FeS_2_ and RC/CNT/0.3FeS_2_/PPy-60 electrodes were charged and discharged 10,000 times at a current density of 15 mA cm^–2^ to measure the cycling performance. Figure 8d shows that the areal capacitance of both electrodes decreases as the number of charge–discharge cycles increases. The RC/CNT/0.3FeS_2_/PPy-60 electrode retained 91.1% of its original capacitance after 10,000 charge/discharge cycles, but the RC/CNT/0.3FeS_2_ electrode could only retain 80%. The dissolution of FeS_2_ is the cause of the poor cycling performance of the RC/CNT/0.3FeS_2_ electrode. The PPy protective layer prevents FeS_2_ dissolution and improves the stability of the RC/CNT/0.3FeS_2_/PPy-60 electrode. In addition, we provided a summary of the capacitance properties of transition metal dichalcogenide (TMD)/conducting polymer/nanocarbon composites-based electrodes as reported in the literature in Table S1.^52−56^ The capacitance value and charge/discharge cycling stability of the RC/CNT/0.3FeS_2_/PPy-60 electrode were superior to those of other TMD composites-based electrodes reported in the literature.

**Figure 9:**
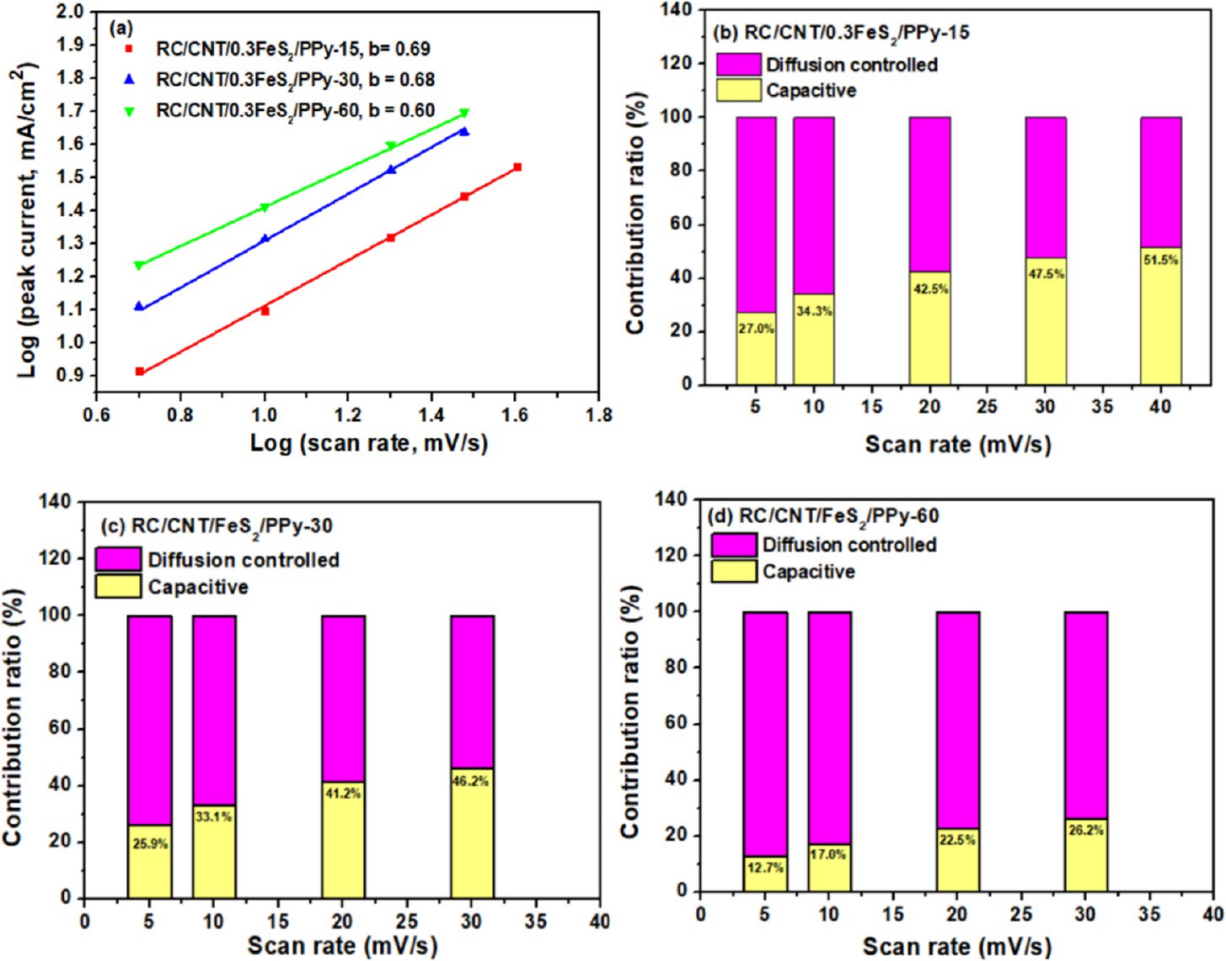
(a) Log(*i*) versus Log(ν) plots for RC/CNT/0.3FeS_2_/PPy electrodes. (b–d) Bar chart showing the % of capacitive contribution of the RC/CNT/0.3FeS_2_/PPy electrodes at various scan rates.

The electrochemical reaction kinetics of the RC/CNT/0.3FeS_2_/PPy electrodes were further studied via CV at multiple scan rates (Figure S12). As the scan rates increase, the oxidation peak shifts to a more positive voltage, and the reduction peak shifts to a more negative voltage. This phenomenon may be attributed to an increase in internal diffusion resistance at high scan rates. The capacitance of an electrode consists of two components. One component involves fast electrochemical processes, such as ion adsorption/desorption (known as the EDLC process), and the rapid faradaic reaction of redox species. This component remains independent of the current density or scan rate. The other component is governed by ion diffusion within the electrode material and the electrolyte. It is crucial to determine which process dominates the electrochemical reaction to provide a more accurate explanation of the electrode performance. During a linear scan with a constant scan rate, the current of an electrode is controlled by the following relationship:^7^
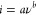
7where *i* is the current, ν is the scan rate, and *a* and *b* are constants. In the case of surface capacitive-controlled processes and the diffusion-controlled process, *b* is equal to 1.0 and 0.5, respectively. In Figure 9a, the oxidation current density of RC/CNT/FeS_2_/PPy-60 is much higher than that of the other electrodes (RC/CNT/FeS_2_/PPy-15 and RC/CNT/FeS_2_/PPy-30). The *b* values are between 0.5 and 0.7, indicating that both diffusion-controlled and surface capacitive-controlled processes exist in the entire electrochemical reaction.^7^ Subsequently, the ratios of the two capacitive mechanism contributions at various scan rates can be calculated (Figure 9b–d). As the scan rate increases, the capacitive contribution exhibits an increasing trend, indicating high-efficiency charge storage. Compared to other electrodes (RC/CNT/FeS_2_/PPy-15 and RC/CNT/FeS_2_/PPy-30), the RC/CNT/FeS_2_/PPy-60 electrode exhibits a higher diffusion-controlled contribution. This suggests that the corresponding redox reactions in the RC/CNT/FeS_2_/PPy-60 electrode are primarily diffusion-controlled processes.

**Figure 10:**
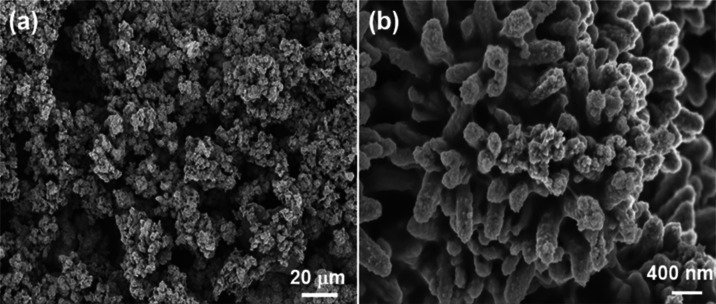
SEM images of the (a) RC/CNT/0.3FeS_2_/PPy-60 (×500) and (b) RC/CNT/0.3FeS_2_/PPy-60 (*×*20,000) electrodes after the 10,000 cycles test.

Figure 10 shows the SEM images of the RC/CNT/0.3FeS_2_/PPy-60 electrode after the 10,000 cycle test. Before the cycle test, the sizes of the PPy/FeS_2_-based particles were less than 1 μm (Figure 5e). The pore sizes of the interstitial spaces between the PPy/FeS_2_-based particles were less than 1 μm (Figure 5f). In contrast, SEM images showed that the PPy/FeS_2_-based particles aggregated into larger particles with diameters of 5–10 μm after the 10,000 cycle test (Figure 9a). The aggregation of the PPy/FeS_2_-based particles results in the formation of larger pore sizes (5–30 μm) of the interstitial spaces, which can be attributed to the physical changes associated with the repeated volumetric shrinking and swelling by the acidic electrolyte. The size of pores in the RC/CNT/0.3FeS_2_/PPy-60 electrode plays the role of ionic transfer channels of the electrolyte. Additionally, Figure S13 displays the FTIR spectra of the RC/CNT/0.3FeS_2_/PPy-60 electrode prior to and subsequent to the 10,000 cycle test. The slight alteration in the absorption peaks of the FTIR spectrum subsequent to the cycle test was ascribed to the marginal degradation of the PPy. After undergoing cycle testing, the capacitance value of the PPy decreases marginally due to degradation and morphological modification.

### 3.4 Electrochemical Properties of the RC/CNT/FeS_2_/PPy Composite Films-Based Symmetric Supercapacitor

**Figure 11:**
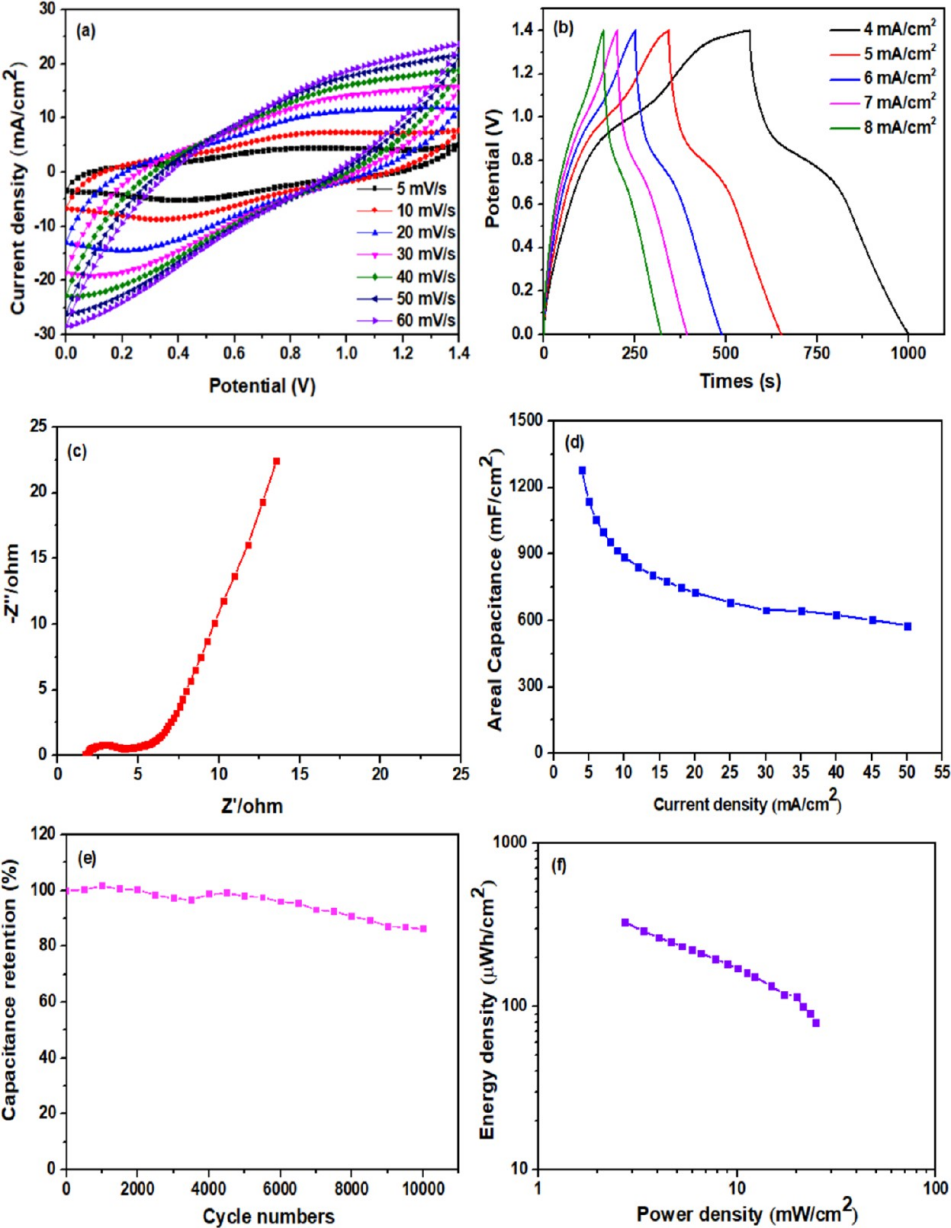
(a) CV plots at different scan rates, (b) GCD plots at different current densities, (c) EIS, (d) areal capacitances plotted with respect to the current density, (e) cycling performance, and (f) Ragone plot of the RC/CNT/0.3FeS_2_/PPy-60 electrode-based symmetric supercapacitor.

A symmetric supercapacitor was assembled to further investigate the practical applications of the RC/CNT/0.3FeS_2_/PPy-60 electrode. The symmetric supercapacitor could achieve a maximum operating voltage of 1.4 V as expected. Figure 11a shows the CV plots of the supercapacitor at different scan rates. The area of the CV curves and the peak current density increased as the scan rate increased, indicating the good rate capability of the supercapacitor. The GCD curves at different current densities, as shown in Figure 11b, clearly show triangular charge–discharge profiles with a slight divergence in linearity, demonstrating the pseudocapacitance contribution in addition to the EDLC behavior. The CV results confirmed this observation. The Nyquist plot of the symmetric supercapacitor fabricated with RC/CNT/0.3FeS_2_/PPy-60 electrodes is shown in Figure 11c. Due to the use of current collectors in the two-electrode system, the *R*_ct_ and *R*_s_ values of the supercapacitor are much smaller than those of the free-standing RC/CNT/0.3FeS_2_/PPy-60 electrode measured in the three-electrode system without current collectors. The areal capacitances of the supercapacitor plotted against the current density are shown in Figure 11d. At 4 mA cm^–2^, a high areal capacitance of 1280 mF cm^–2^ was achieved, and it could still maintain 577.9 mF cm^–2^ when the current density was increased to 50 mA cm^–2^, corresponding to the superior rate capability of the assembled supercapacitor device. The high rate capability was related to the homogeneously distributed FeS_2_ microspheres on the RC/CNT porous substrate, which enhances the electrical conductivity and the electrochemically active surface area for effective electrolyte ion accessibility.

The symmetric supercapacitor device exhibited excellent cycle life stability with a capacitance retention of 86.2% of the initial capacitance after 10,000 cycles at 40 mA cm^–2^ when tested at a wide voltage window of 1.4 V (Figure 11e). From the GCD curves, the energy density (*E*, μW h cm^–2^) and power density (*P*, μW cm^–2^) of the symmetric supercapacitor were calculated using eqs 3 and 4, respectively. The Ragone plot in Figure 11f shows the energy density as a function of the power density. The supercapacitor device delivered a maximum energy density of 329.7 μWh cm^–2^ at a power density of 2.7 mW cm^–2^ and a maximum power density of 24.9 mW cm^–2^ at an energy density of 79.5 μWh cm^–2^. The following factors contributed to the improved electrochemical performance of the symmetric supercapacitor based on RC/CNT/0.3FeS_2_/PPy-60 electrodes: (1) Highly conductive and porous RC/CNT substrate with uniform distribution of FeS_2_ microspheres, which provides a large electrochemically active surface area for efficient ion adsorption from the electrolyte; (2) The FeS_2_ microspheres, which consist of agglomerates of seed-like nanostructures, help to enhance the surface redox reaction of FeS_2_ with intercalated Na+ ions; (3) The incorporation of PPy coating on the RC/CNT/0.3FeS_2_ composite improves the overall electrical conductivity of the RC/CNT/0.3FeS_2_/PPy-60 composite and prevents the dissolution of FeS_2_; (4) The enhanced voltage of 1.4 V achieved for the symmetric supercapacitor results in high energy density and power density.

## 4 Conclusions

The study highlights the utilization of a composite material comprising RC/CNT with remarkable conductivity and porosity as a host for anchoring active materials FeS_2_ and PPy. The FeS_2_ microflowers contribute to an open porous structure, facilitating electrolyte ion access to the entire active material surface, thereby optimizing material utilization. The synergistic effects arising from the combination of the high active surface area of CNTs and the redox-active nature of FeS_2_ microflowers and PPy likely contribute to the exceptional performance observed in the RC/CNT/FeS_2_/PPy composite film electrodes. Furthermore, the protective layer of PPy serves to prevent FeS_2_ dissolution, thereby enhancing the overall conductivity and stability of the electrode. The electrode denoted as RC/CNT/0.3FeS_2_/PPy-60 exhibited the highest areal capacitance and superior cycle life stability among the tested compositions. Moreover, when integrated into a symmetrical supercapacitor device, the RC/CNT/0.3FeS_2_/PPy-60 composite electrodes demonstrated exceptional areal capacitance, energy density, power density, and cycling stability, especially at a high cell voltage of 1.4 V. These findings underscore the potential of the developed composite material for advanced energy storage applications.

## Supporting Information Available

FTIR and XPS spectra; Tauc-plots, AC-2 low energy photoelectron spectra, and energy diagram; XRD patterns of RC/CNT/FeS_2_/PPy films; SEM images of RC/CNT; HRTEM of FeS_2_; EDS images of the RC/CNT/0.3FeS_2_/PPy-60; TGA curves of the RC/CNT/0.3FeS_2_/PPy films; N_2_ adsorption–desorption isotherms of RC/CNT/FeS_2_ composites; specific capacitances of the RC/CNT/FeS_2_/PPy electrodes (PDF)
