# Supplementary material for: Iron Sulfide Microspheres Supported on Cellulose-Carbon Nanotube Conductive Flexible Film as an Electrode Material for Aqueous-Based Symmetric Supercapacitors with High Voltage
Source: ACS Omega. 2024 Jun 6;9(24):26582–95. doi: 10.1021/acsomega.4c03232 (PMC11191093; doi:10.1021/acsomega.4c03232)
Supplement: Supplementary file 1 — ao4c03232_si_001.pdf [file ao4c03232_si_001.pdf]

Supporting Information

Iron sulfide microspheres supported on cellulose-carbon nanotube conductive flexible film as an electrode material for aqueous-based symmetric supercapacitors with high voltage

Jincy Parayangattil Jyothibasu[a], You-Ching Tien[a], Zi-Ting Chen[a], Hongta Yang[a], Tzu Hsuan Chiang[b], Ahmed F.M. EL-Mahdy[c], and Rong-Ho Lee[*a,d]

[a] Department of Chemical Engineering, National Chung Hsing University, Taichung, 402, Taiwan

[b] Department of Energy Engineering, National United University, Miaoli, 360302, Taiwan.

[c] Department of Materials and Optoelectronic Science, National Sun Yat-Sen University, Kaohsiung, 80424, Taiwan

[d.] Department of Chemical Engineering and Materials Science, Yuan Ze University, Taoyuan City 320, Taiwan

* To whom all correspondence should be addressed.

Tel.: 886-4-22854308; Fax: 886-4-22854734

E-mail: rhl@nchu.edu.tw

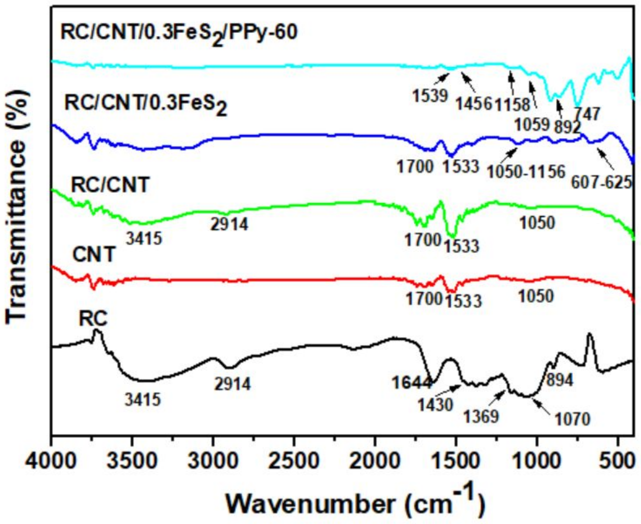

Figure S1. FTIR spectra of RC, CNT, RC/CNT, RC/CNT/0.3FeS_2, and RC/CNT/0.3FeS_2/PPy-60 composite films.

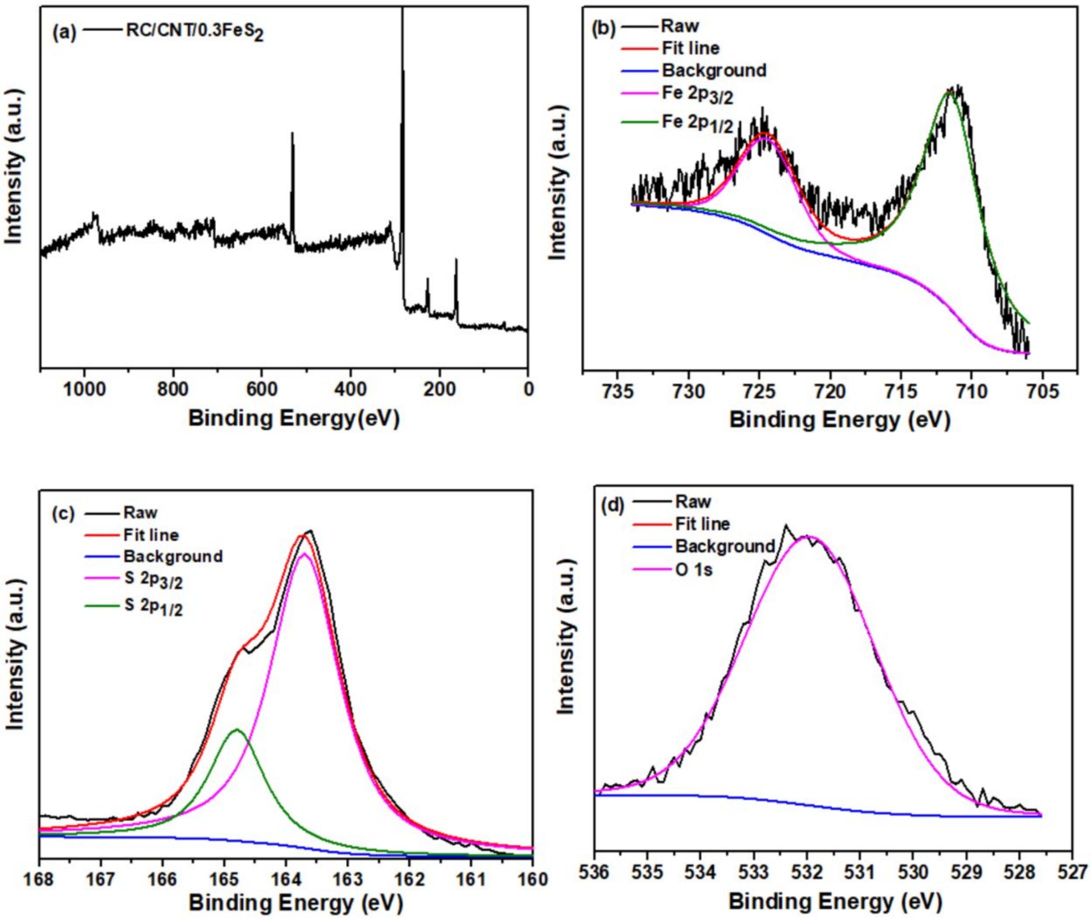

Figure S2. (a) XPS survey spectrum of RC/CNT/0.3FeS_2 composite film. XPS spectra (b) Fe 2p, (c) S 2p, and (d) O 1s binding energies of RC/CNT/0.3FeS_2 composite film.

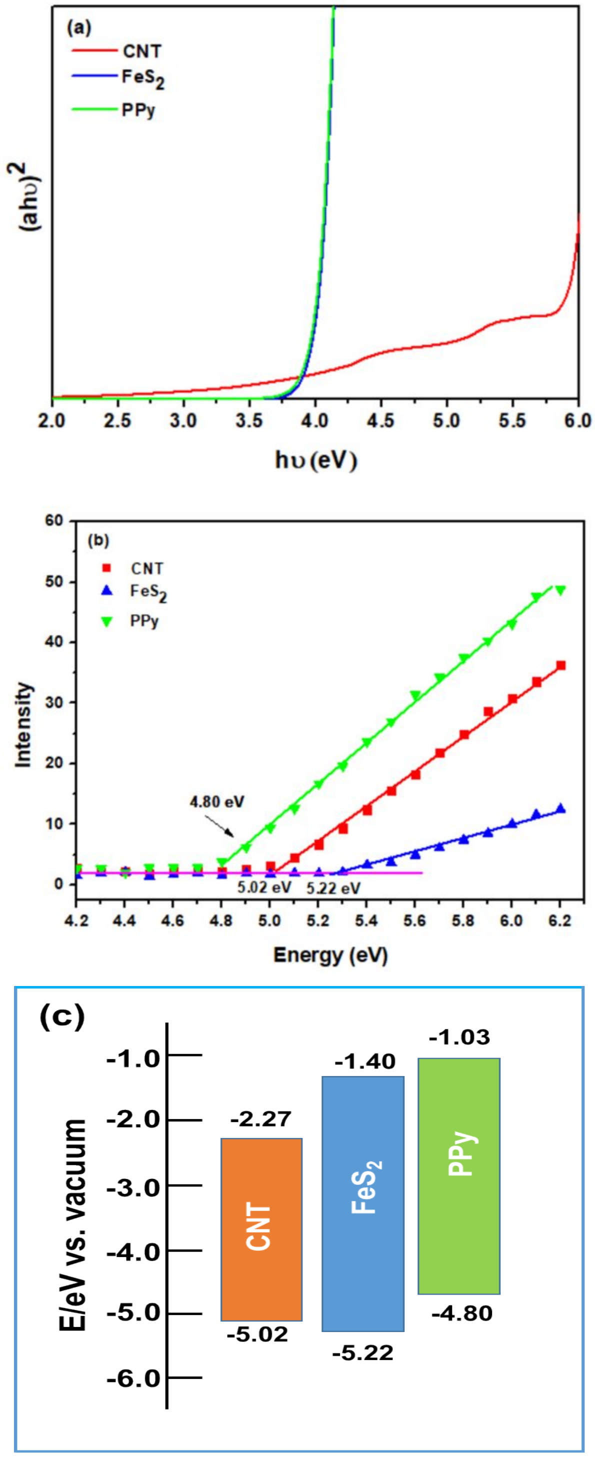

Figure S3. The tauc-plots, AC-2 low energy photoelectron spectra, and energy diagram of the CNT, FeS_2, and PPy materials.

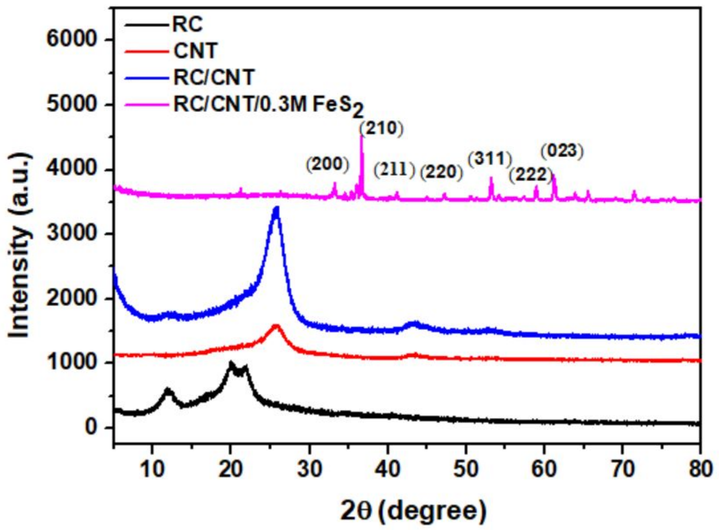

Figure S4. XRD patterns of RC, CNT, RC/CNT, and RC/CNT/0.3FeS_2 composite films.

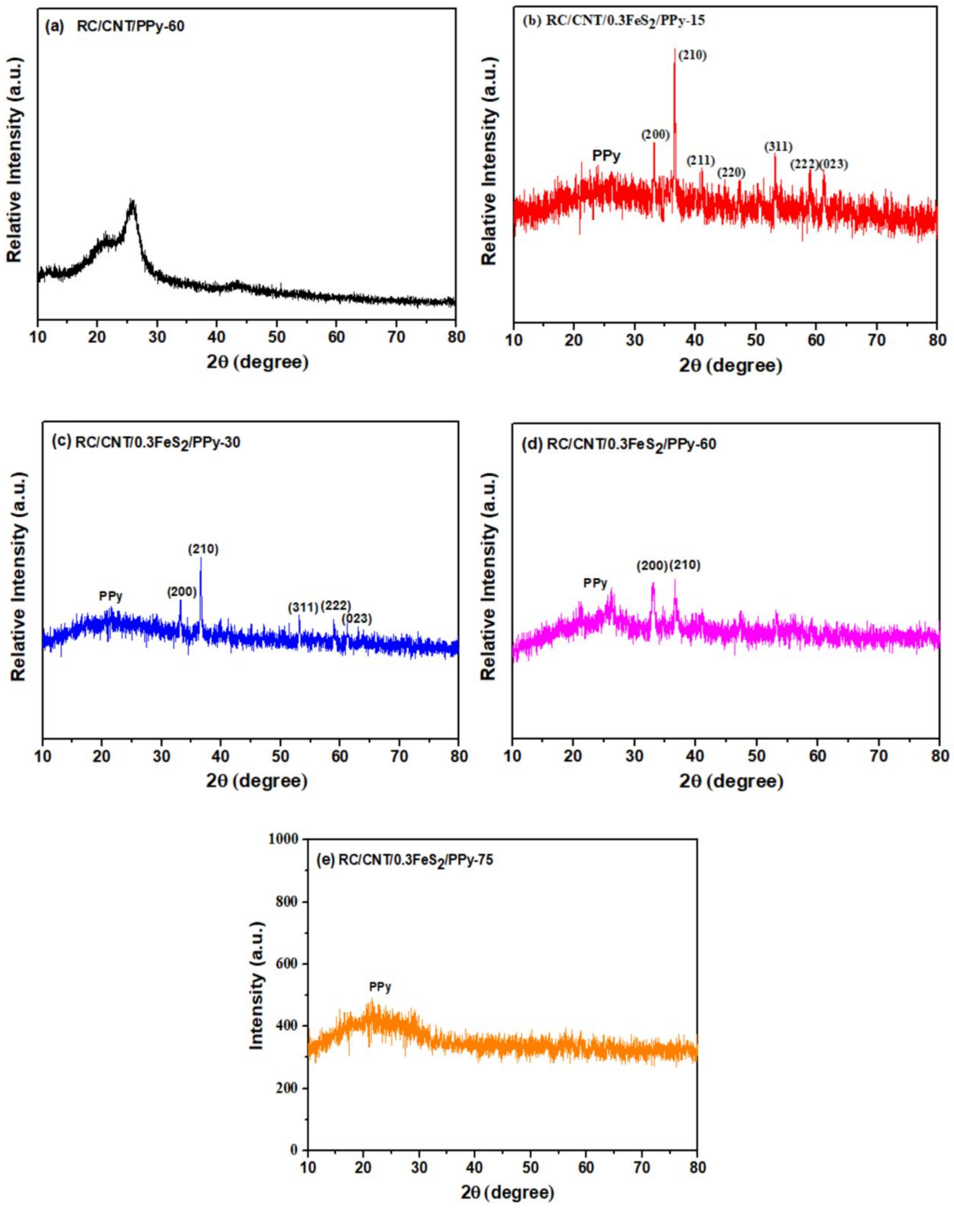

Figure S5. XRD patterns of RC/CNT/PPy-60, RC/CNT/0.3FeS_2/PPy-15, RC/CNT/0.3FeS_2/PPy-30, RC/CNT/0.3FeS_2/PPy-60, and RC/CNT/0.3FeS_2/PPy-75 composites.

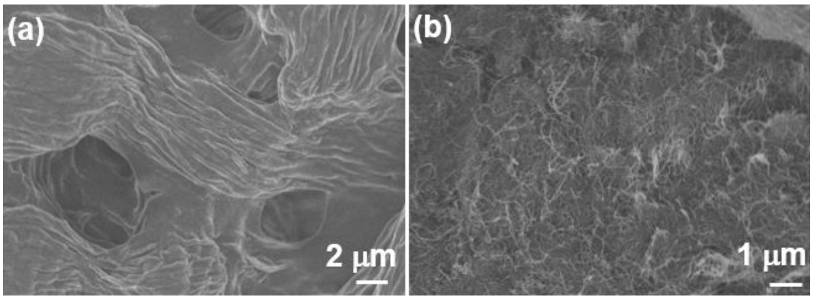

Figure S6. SEM images of (a) RC and (b) RC/CNT films.

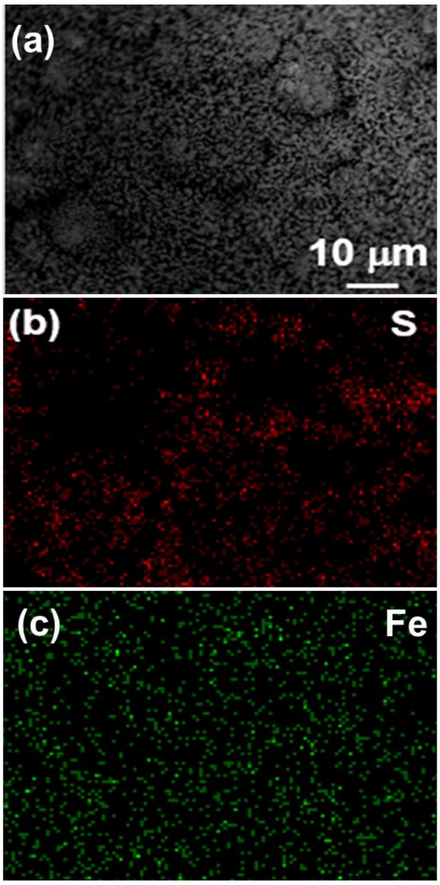

Figures S7. (a) SEM and ((b),(c)) EDX images of the (b) S, and (c) Fe elements in the RC/CNT/0.3FeS_2 composite.

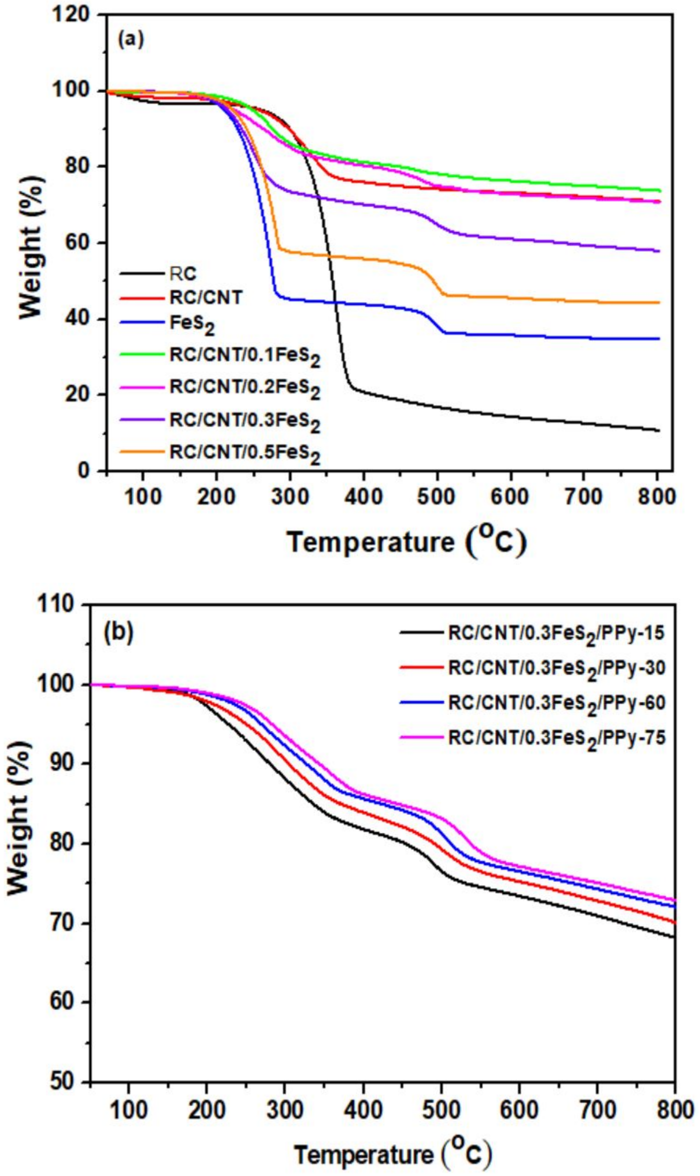

Figure S8. TGA curves of (a) RC, RC/CNT, FeS_2, RC/CNT/0.1FeS_2, RC/CNT/0.2FeS_2, RC/CNT/0.3FeS_2, and RC/CNT/0.5FeS_2; (b) RC/CNT/0.3FeS_2/PPy-15, RC/CNT/0.3FeS_2/PPy-30, RC/CNT/0.3FeS_2/PPy-60, and RC/CNT/0.3FeS_2/PPy-75 composites.

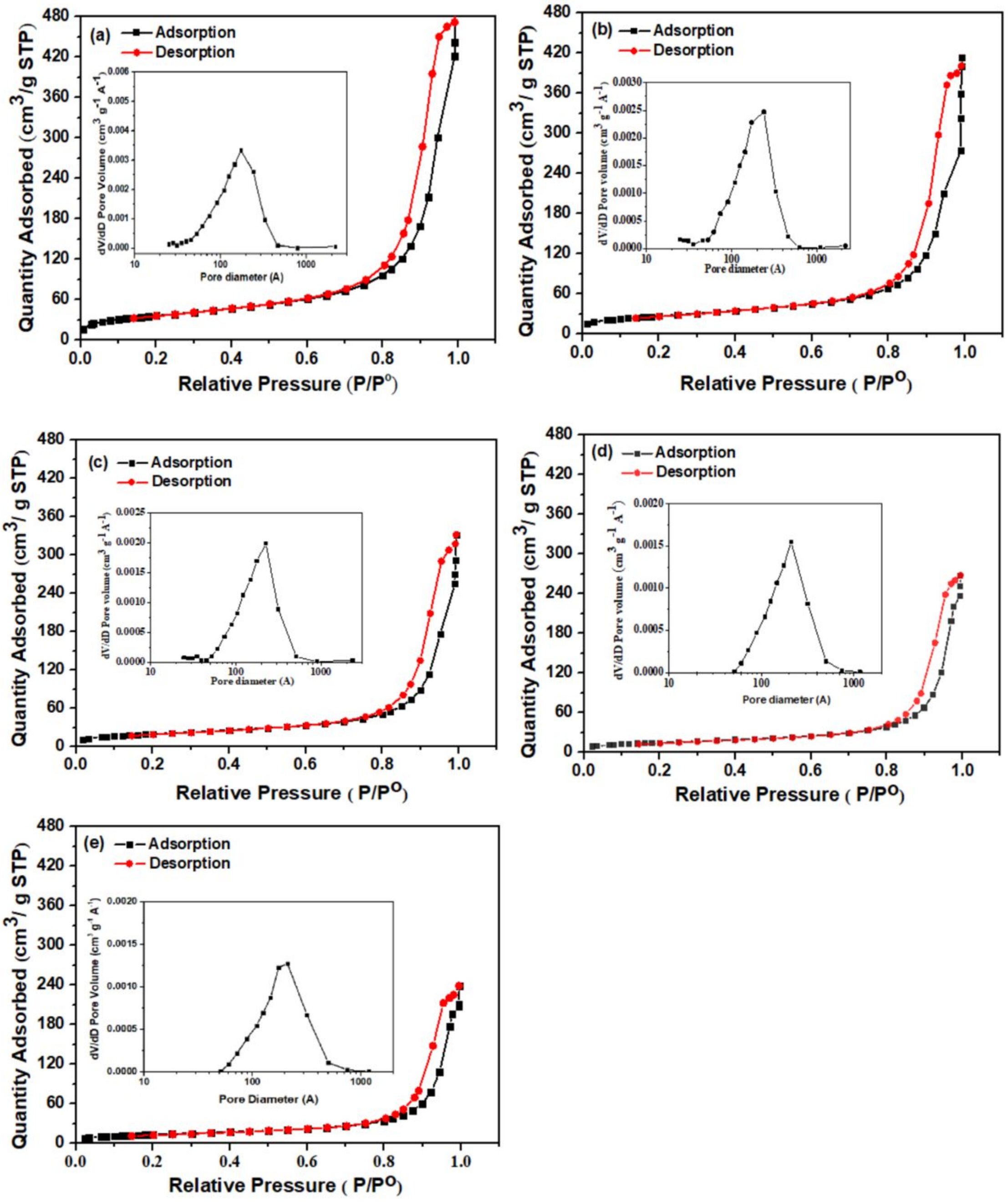

Figure S9. N_2 adsorption-desorption isotherms and pore size distributions of (a) RC/CNT, (b) RC/CNT/0.1FeS_2, (c) RC/CNT/0.2FeS_2, (d) RC/CNT/0.3FeS_2, and (e) RC/CNT/0.5FeS_2 composites films.

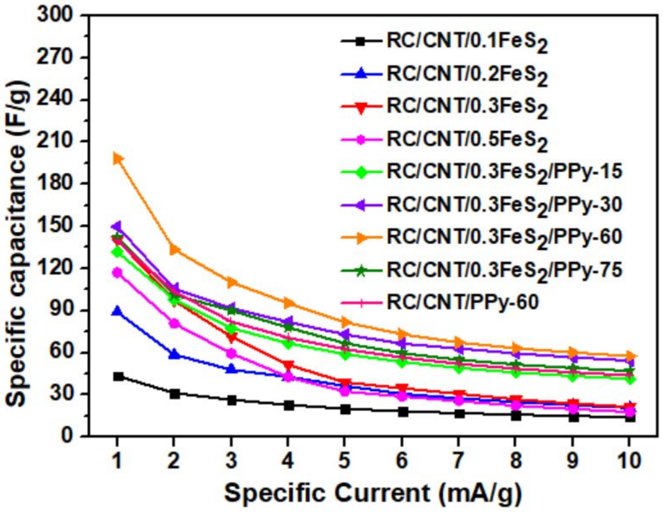

Figure S10. Specific capacitances of the RC/CNT/0.1FeS_2, RC/CNT/0.2FeS2, RC/CNT/0.3FeS_2, RC/CNT/0.5FeS_2, RC/CNT/0.3FeS_2/PPy-15, RC/CNT/0.3FeS_2/PPy-30, RC/CNT/0.3FeS_2/PPy-60, RC/CNT/0.3FeS_2/PPy-75, and RC/CNT/PPy-60 composites plotted with respect to the specific current.

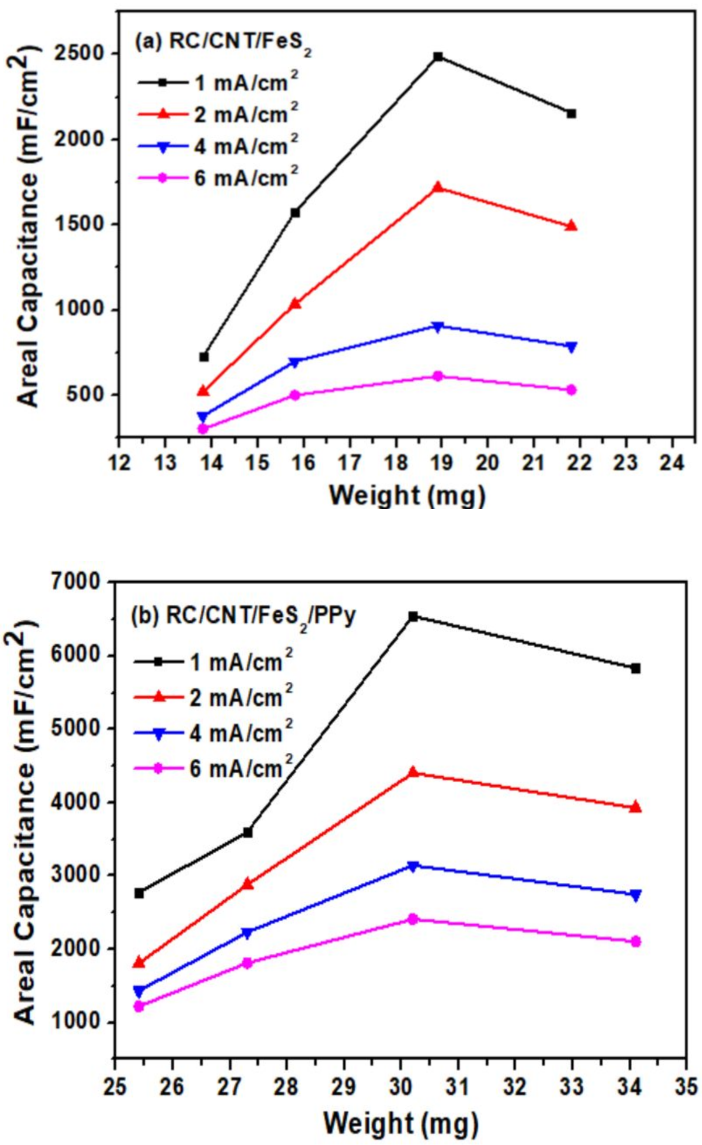

Figure S11. The areal capacitance values plotted against the weight of the RC/CNT/FeS_2 and RC/CNT/FeS_2/PPy electrodes.

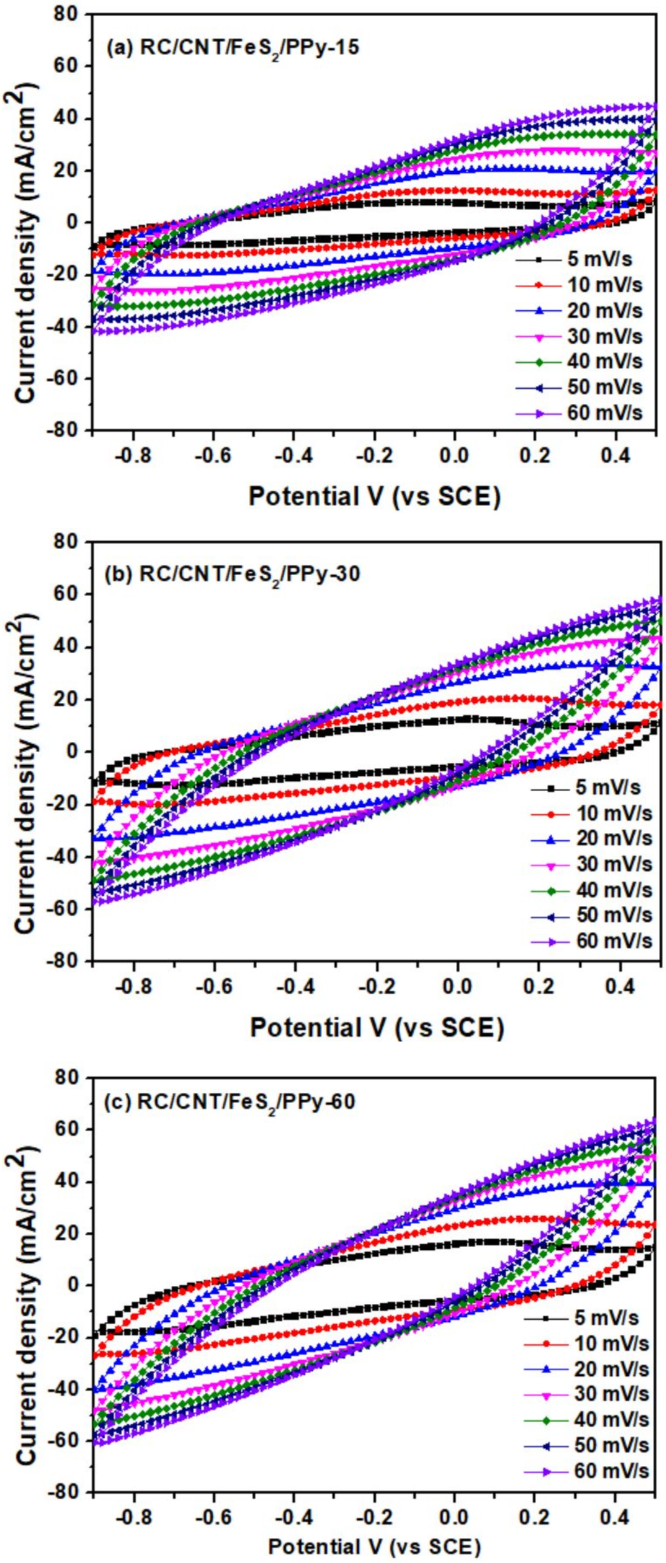

Figure S12. CV plots at different scan rates of (a) RC/CNT/FeS_2/PPy-15, (b) RC/CNT/FeS_2/PPy-30, and (c) RC/CNT/FeS_2/PPy-60.

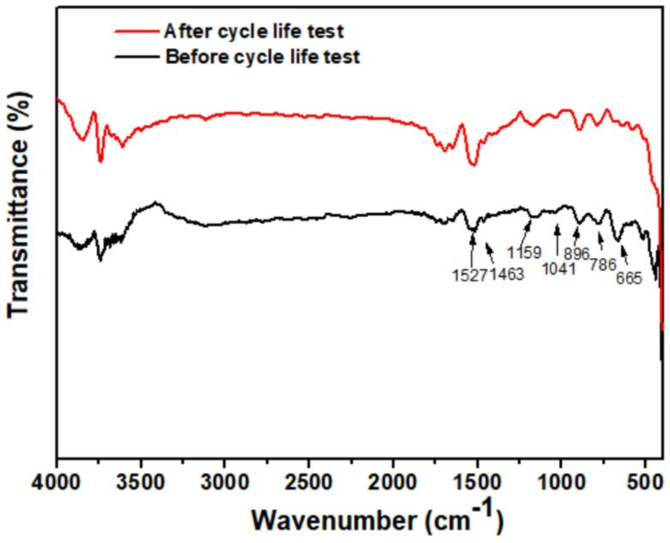

Figure S13. FTIR spectra of the RC/CNT/0.3FeS_2/PPy-60 electrode before and after the 10,000 cycle test.

Table S1. The capacitance properties of the transition metal dichalcogenide (TMD)/conducting polymer/nano-carbon composites-based electrodes.

Electrode	Electrolyte	C_A (mF/cm^2)	C_m (F/g)	Cycling test stability (%)	References
RC/CNT/FeS_2/PPy	1 M Na_2SO_4 aqueous	1280.0	198.29	91.1 (10000 cycles)	This work
Ni_3S_2@Ni/CC	6.0 M KOH aqueous	2420.0	---	91.5 (5000 cycles)	[51]
rGO@Ni_3S_2/CC	6.0 M KOH aqueous	----	477.37	88.0 (5000 cycles)	[52]
PPy/CuS/BC	2.0 M NaCl aqueous	----	580.00	73.0 (300 cycles)	[53]
MoS_2/PPy/CNFs	----	734.0	---	84.0 (after 2000 cycles)	[54]
PPy/MoS_2/CC	5 M LiCl aqueous	1150.4	---	87.2% (5000 Cycles)	[55]
